# Sleep-Gated Adaptation: A Conceptual Framework Linking Exercise-Induced Adaptive Consolidation to Interindividual Variability in Training Response

**DOI:** 10.3390/ijms27198811

**Published:** 2026-10-01

**Authors:** Teodora Dominteanu, Amelia Elena Stan, Andreea Voinea

**Affiliations:** 1 Department of Physical Education and Sport, Faculty of Agrifood and Environmental, Bucharest University of Economic Studies, 010374 Bucharest, Romania; andreea.voinea@defs.ase.ro; 2 Faculty of Physical Education and Sport, Romanian-American University, Exhibition Boulevard No. 1B, Sector 1, 012101 Bucharest, Romania; amelia.stan@rau.ro

**Keywords:** exercise adaptation, sleep, skeletal muscle, recovery, interindividual variability, immune remodeling, heart-rate variability, autonomic regulation, training response, consolidation

## Abstract

Exercise-induced molecular signaling is transient, resolving within hours, whereas the structural and functional adaptations to repeated training develop over weeks to months. The processes that convert signaling into durable adaptation, the conditions that best support this conversion, and the sources of interindividual variability in training outcomes have largely been studied separately. This review proposes a conceptual framework for integrating these approaches. Adaptive Consolidation is introduced as an organizing concept for this intermediate transition, synthesized as a multi-filter cascade spanning post-translational modification, transcription, protein turnover, chromatin remodeling, immune-cell reprogramming, and autonomic remodeling. Sleep-Gated Adaptation (SGA) is proposed as a physiological gate for this transition; evidence indicates that sleep dependence is domain-conditional rather than uniform, strongest for protein-synthetic and motor-memory domains, and detectable in immune and autonomic domains only under specific combinations of exercise damage, sleep-loss severity, and readout timing. Adaptive Consolidation Efficiency (ACE) is proposed as an organizing label for individual-specific determinants—genetic, epigenetic, baseline-biological, environmental, and prescription-related—that jointly explain variability in training response. Neither the SGA nor the ACE is proposed as an established mechanism, validated biomarker, or quantitative index; both are conceptual constructs integrating heterogeneous but convergent evidence. The framework was translated into seven falsifiable predictions, a biomarker panel of validated readouts, and minimum design requirements, including falsification criteria, for future testing.

## 1. Introduction

### 1.1. Background and Rationale

Exercise is among the most extensively characterized physiological stimuli for driving long-term biological adaptation, and the intracellular signaling networks that transduce a single exercise bout—AMP-activated protein kinase (AMPK), mechanistic target of rapamycin complex 1 (mTORC1), peroxisome proliferator-activated receptor gamma coactivator-1α (PGC-1α), and associated calcium-dependent and redox-sensitive pathways—have been described in considerable mechanistic detail [1,2].

However, the molecular signals activated during and immediately after a single exercise bout are transient, typically resolving within hours, whereas the structural and functional adaptations associated with repeated training develop over weeks to months and persist well after the initiating signals disappear [3,4]. This temporal dissociation is well documented at the level of individual signaling pathways; however, the biological processes responsible for converting a transient molecular pulse into a stable physiological trait have rarely been organized within a single conceptual framework spanning multiple tissue systems.

A parallel, largely separate literature has established that several components of this putative consolidation process—anabolic hormone secretion, myofibrillar protein synthesis, mitochondrial respiratory adaptation, and motor-skill retention—are measurably impaired by experimental sleep restriction [5,6,7,8,9,10]. This evidence is rarely framed as bearing on the signaling-to-adaptation transition specifically; sleep is more commonly treated as one input to general postexercise recovery, alongside nutrition and training-load management, rather than as a physiological gate that determines whether a given bout of adaptive signaling is successfully consolidated into a durable change.

Third, a separate literature addresses why individuals exposed to standardized training programs show markedly heterogeneous outcomes. Foundational work in the HERITAGE Family Study established a substantial heritable component to trainability [11,12], but subsequent reanalysis showed that response is trait-specific rather than reflecting a stable global phenotype [13,14], and that a meaningful share of apparent non-response instead reflects epigenetic state [15,16], training-modality mismatch [17], or measurement noise [18] rather than a fixed biological ceiling. This literature has also developed largely independently of the signaling-and-consolidation literature described above.

These three bodies of evidence—exercise-induced molecular signaling, sleep-dependent physiological recovery, and interindividual variability in training response—are conceptually connected, as each describes a stage or determinant of the same underlying process, namely, the conversion of a transient exercise stimulus into a durable physiological trait. However, to our knowledge, they have not been integrated within a single systems-level framework. This review addresses this gap by proposing Adaptive Consolidation as an organizing concept for the intermediate biological transition, Sleep-Gated Adaptation (SGA) as a candidate physiological gate governing that transition, and Adaptive Consolidation Efficiency (ACE) as an organizing label for the individual-specific determinants of how completely that transition occurs.

### 1.2. Objectives

The specific objectives of this review are as follows: (1) to synthesize evidence distinguishing transient exercise-induced molecular signaling from durable physiological adaptation and to organize the intervening biological processes under a single conceptual framework, Adaptive Consolidation (Section 2.1 and Section 2.2); (2) to evaluate, domain by domain, the evidence that sleep provides a physiological gate for this consolidation process and to characterize the boundary conditions under which this gating is and is not detectable (Sleep-Gated Adaptation; Section 2.3); (3) to synthesize the principal determinants of interindividual variability in training adaptation and to organize them as candidate determinants of consolidation efficiency (Adaptive Consolidation Efficiency; Section 2.4); and (4) to translate the resulting conceptual framework into a set of falsifiable predictions, a candidate biomarker panel, and minimum study-design requirements suitable for future empirical testing (Section 3).

### 1.3. Guiding Research Questions

Q1. Does exercise generate transient molecular signaling that should be conceptually and empirically distinguished from the process of physiological adaptation itself, and which biological mechanisms mediate the transition between the two?
Q2. Under which physiological conditions is this transition most effectively supported? Does sleep function as a necessary, permissive gate for consolidation, or merely as a correlate among several general post-exercise recovery factors?
Q3. Do all components of the proposed consolidation process—endocrine, immune, protein turnover, neural, mitochondrial, circadian, metabolic, and autonomic—show equivalent evidence of sleep dependence, or is this dependence conditional and domain-specific?
Q4. Why do individuals exposed to comparable exercise stimuli and comparable sleep conditions nonetheless show markedly different long-term adaptive outcomes, and which determinants jointly account for this variance?
Q5. Can the resulting conceptual framework be operationalized into predictions that are, in principle, falsifiable rather than remaining a purely narrative synthesis?

### 1.4. Scope and Manuscript Structure

This review is conceptual and hypothesis-generating. Neither Sleep-Gated Adaptation nor Adaptive Consolidation Efficiency is proposed as an established mechanism, a validated biomarker, or a quantitative index; both are proposed as organizing constructs intended to integrate mechanistically heterogeneous but empirically convergent evidence, in the sense clarified further in Section 2.3 and Section 2.4. Literature was identified using a targeted, question-driven strategy rather than a PRISMA-based systematic review protocol, as detailed in the Literature Review Strategy and Scope subsection immediately following this Introduction.

The remainder of this manuscript is organized as follows. Section 2 develops the conceptual framework across four parts: the distinction between exercise-induced adaptive information and adaptation itself (Section 2.1), the mechanistic cascade underlying Adaptive Consolidation, including its immune and autonomic components (Section 2.2), the evidence for Sleep-Gated Adaptation and its domain-specific boundary conditions (Section 2.3), and the evidence for interindividual variability underlying Adaptive Consolidation Efficiency (Section 2.4). Section 3 translates this framework into seven falsifiable predictions, a candidate biomarker panel, and the minimum design requirements for future empirical testing.

### 1.5. Literature Review Strategy and Scope

Evidence for this review was identified using Elicit, an AI-assisted literature discovery and synthesis tool that queries a large indexed corpus of scholarly publications rather than a fixed-database systematic search protocol. The approach was targeted and question-driven: rather than running broad keyword strings across a small set of databases and screening the resulting hit list, each conceptual claim requiring evidentiary support was decomposed into a specific, mechanistically and temporally framed question. For example, rather than asking whether sleep restriction “affects” postexercise recovery in general, questions specified a named physiological readout and a defined postexercise window (e.g., whether sleep restriction alters the kinetics of postexercise interleukin-6 across a 0–48 h window). Elicit was queried with each question individually, and the papers it returned were evaluated for direct relevance to the question, for mechanistic or temporal specificity—serial or interventional data were prioritized over single-timepoint or purely observational findings—and for coherence with directly adjacent evidence already retained.

**Figure 1:**
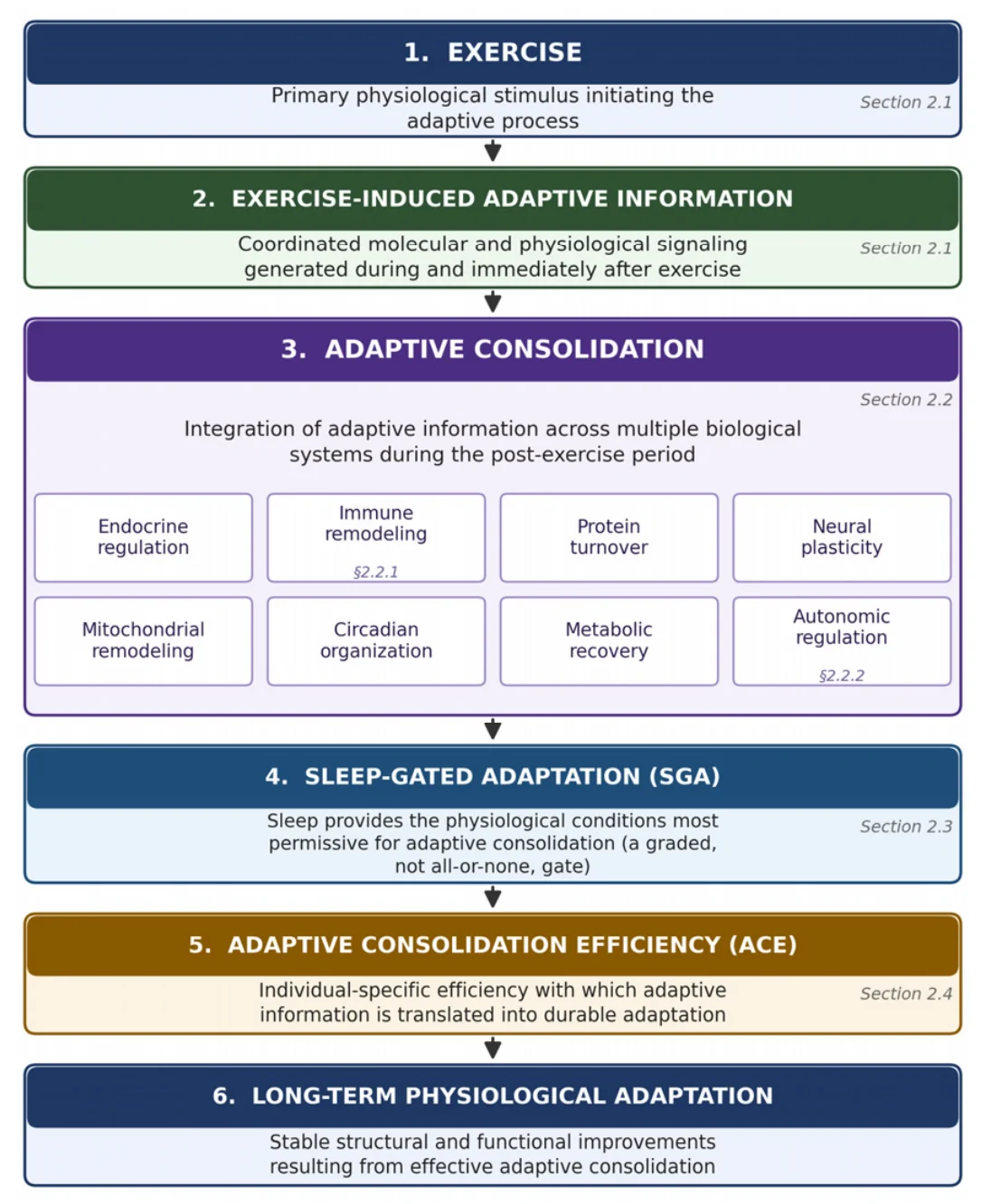
The SGA–ACE conceptual framework of exercise adaptation. Exercise generates adaptive biological information through coordinated molecular and physiological signaling (Section 2.1). This information subsequently enters a phase of Adaptive Consolidation (Section 2.2), during which multiple biological systems contribute to its stabilization and integration. Sleep-Gated Adaptation (SGA, Section 2.3) represents the biological principle that sleep provides the most favorable physiological conditions for adaptive consolidation, while Adaptive Consolidation Efficiency (ACE, Section 2.4) describes the overall effectiveness with which adaptive information is ultimately translated into durable physiological adaptation, as shaped by genetic, epigenetic, baseline-biological, environmental, and training-prescription factors.

This process was iterative rather than a single search. An initial round of questions supported the core conceptual claims developed in Section 2.1, Section 2.2, Section 2.3 and Section 2.4: the distinction between exercise-induced signaling and durable adaptation, the mechanistic cascade underlying Adaptive Consolidation, and the sleep dependence and interindividual variability evidence underlying Sleep-Gated Adaptation (SGA) and Adaptive Consolidation Efficiency (ACE). During the internal review, a discrepancy was identified between the domains depicted in the summary figure (Figure 1), which listed eight consolidation domains, and the narrative text, which at that stage provided direct evidentiary support for only six. A second, targeted round of questions was then formulated specifically to source direct evidence for the two underrepresented domains—immune remodeling and autonomic regulation—including explicit sub-questions on whether each domain shows the same transient perturbation kinetics, durable structural remodeling, and conditional sleep dependence already established for the muscle-signaling and endocrine domains. Where the resulting evidence for a given interaction was genuinely mixed, both positive and null findings were retained and reported, and the boundary conditions distinguishing them (exercise damage or duration, sleep-loss severity and duration, and readout timing) were made explicit in the narrative rather than being resolved by selective citation.

This review did not follow the PRISMA-based systematic review protocol. No fixed set of databases, Boolean query strings, or pre-registered inclusion/exclusion criteria were applied; no independent dual-reviewer screening was conducted; and the exact search dates and corpus version accessed through Elicit were not systematically logged during the drafting process. Accordingly, the evidence base underlying this review should be understood as a structured, mechanistically prioritized narrative synthesis rather than an exhaustive or reproducible systematic review, consistent with the conceptual and hypothesis-generating aims of this manuscript.

The resulting evidence base is heterogeneous in terms of study type and species. Priority was given throughout to human experimental and interventional studies with serial or time-resolved measurements; where a specific mechanistic claim rests primarily or exclusively on rodent or cellular evidence, this is stated explicitly in the surrounding text (see, for example, Section 2.2.1 and Section 2.2.2). The evidence base also includes a small number of preprints and conference or meeting abstracts (e.g., several FASEB Journal supplement abstracts, one bioRxiv preprint, and one Medicine & Science in Sports & Exercise conference abstract), which have not undergone the same peer-review process as full research articles and should be weighted accordingly when the claims they support are evaluated.

We acknowledge that this targeted, question-driven approach does not permit an independent reader to reconstruct the boundaries of the search or to determine what proportion of the relevant literature, including literature that might contradict the framework proposed here, was not retrieved. This is a genuine limitation of the evidentiary basis of this review, not merely a matter of reporting style, and it is revisited explicitly in Section 4.3.

We further acknowledge the methodological concern that the sequence described above—identifying a gap in evidentiary coverage relative to an already-drafted figure, and then searching specifically to fill that gap—risks evidence being sought to complete a pre-specified architecture rather than the architecture emerging neutrally from the evidence. We did not have a mechanism, such as a pre-registered protocol or blinded search, to fully exclude this risk. As a partial mitigation, the second round of questions was formulated to ask neutrally whether each domain showed the same three properties (transient perturbation kinetics, durable structural remodeling, and conditional sleep dependence) rather than to ask whether it confirmed the framework, and the resulting evidence for both domains included boundary conditions and partial null findings rather than uniformly positive support (Section 2.2.1 and Section 2.2.2, and Section 2.3). Nonetheless, a neutral, pre-registered evidence-identification protocol, applied prospectively across all eight domains rather than reactively to the two initially underrepresented, would be necessary to fully exclude confirmation-driven evidence selection; we identify this as an explicit priority for future replication in Section 4.3.

## 2. Conceptual Framework

### 2.1. Exercise-Induced Adaptive Information

Exercise is universally recognized as the primary physiological stimulus that drives long-term adaptation. Each exercise bout transiently perturbs biological homeostasis through coordinated mechanical, metabolic, neural, endocrine, inflammatory, and redox stimuli, thereby activating a complex network of intracellular signaling pathways that regulate the cellular plasticity. Over the past two decades, substantial progress has been made in characterizing these molecular responses, and signaling networks such as AMP-activated protein kinase (AMPK), mechanistic target of rapamycin complex 1 (mTORC1), peroxisome proliferator-activated receptor gamma coactivator-1α (PGC-1α), calcium-dependent signaling, mitogen-activated protein kinases (MAPKs), and redox-sensitive transcriptional pathways have been established as major regulators of exercise-induced adaptation. Collectively, these pathways constitute the biological interface through which exercise communicates physiological demands to tissues and initiates adaptive processes [1,2].

Despite this detailed understanding of exercise-induced signaling, acute molecular activation should not be equated with physiological adaptation. Most signaling events elicited by exercise are transient, occurring over minutes to hours, before progressively returning to baseline. In contrast, the structural and functional adaptations associated with repeated training, including skeletal muscle hypertrophy, mitochondrial biogenesis, angiogenesis, extracellular matrix remodeling, improved neuromuscular function, and enhanced metabolic capacity, develop progressively over weeks or months and often persist long after the initiating molecular signals have disappeared. The clearest human demonstration of this temporal dissociation comes from a single-bout time-course study in which a subset of exercise-responsive genes rose sharply after the first training session and returned to baseline within hours, while mitochondrial proteins (cytochrome c, COXIV, citrate synthase activity) accumulated progressively across two weeks of daily cycling, alongside a 17.5% increase in VO_2_peak, despite the mitochondrial DNA copy number remaining unchanged at that time point [4]. This dissociation demonstrates that exercise-induced signaling and long-term physiological adaptation represent distinct biological phenomena that operate on different timescales [1,3,4].

Recent advances in transcriptomics, proteomics, and epigenomics have further strengthened this distinction. Comprehensive molecular profiling has demonstrated that a single bout of exercise induces widespread but predominantly transient changes in gene expression, protein abundance, metabolite concentrations, and signaling activity across multiple organs and tissues. In the canonical time-course description, a single bout of one-legged knee-extensor exercise triggers 3- to 20-fold increases in the transcription of metabolic genes (*UCP3*, *PDK4*, *HO-1*) that peak 1–2 h into recovery before returning to baseline within roughly a day, consistent with the conclusion that the cumulative effect of repeated transient transcriptional pulses—rather than any single bout—provides the kinetic basis for training-induced cellular adaptation [3]. Multi-tissue profiling across nine omics platforms in a large endurance-training model further shows that this transient, pulsatile architecture is not confined to skeletal muscle; training-time-resolved changes occur across immune, metabolic, stress-response, and mitochondrial pathways in multiple organs, with tissue- and sex-specific kinetics [19]. Importantly, only a fraction of these acute molecular responses ultimately contribute to stable physiological remodeling. Many biological responses resolve without measurable long-term consequences, whereas others are progressively retained and reinforced through repeated training stimuli, ultimately contributing to a durable adaptation [3,4,19].

Collectively, these observations suggest that exercise should be viewed as generating exercise-induced adaptive information rather than adaptation. Within the present framework, exercise-induced adaptive information refers to an ensemble of regulatory instructions encoded by exercise-activated signaling networks that collectively specify the direction of subsequent biological remodeling. This terminology does not imply the existence of a distinct biological entity; rather, it provides a conceptual framework for integrating the diverse molecular signals initiated by exercise into a unified representation of the adaptive information available for subsequent physiological adaptation. Mechanical loading, energetic stress, calcium signaling, endocrine activation, inflammatory mediators, reactive oxygen species, and neural inputs encode complementary biological information regarding the physiological demands imposed by exercise. Notably, the informative content of this signaling is not a simple linear function of the exercise intensity. Phospho-proteomic and kinetic modeling studies indicate that key kinase/phosphatase interactions (e.g., AMPK and CaMKII) behave as trigger-like transients concentrated within the first minutes of a bout, shaped by feedback among kinases and phosphatases rather than by a monotonic dose–response relationship [2].

This perspective provides an important conceptual distinction between the initiation and completion of adaptation. Although exercise-induced signaling defines the biological potential for adaptation, the magnitude of the initial molecular response alone cannot fully explain the considerable variability observed in long-term physiological outcomes. Individuals exposed to comparable exercise stimuli frequently demonstrate markedly different adaptive responses despite broadly similar acute molecular activation, suggesting that biological events occurring after signal generation play a decisive role in determining whether adaptive information is ultimately translated into durable physiological remodeling [1,20]. Consistent with this, comparisons between endurance-trained athletes and previously sedentary individuals undergoing an 8-week training program show that the genes acutely activated by a single intense bout overlap only partially with the genes that distinguish the trained from the untrained transcriptomic signature, indicating that the long-term adaptive phenotype is not simply the accumulated echo of the acute response but emerges from repeated signaling layered onto a progressively remodeled biological substrate [20]. In practical terms, this distinction implies that the presence of a strong acute signaling response to a training session—for example, pronounced post-exercise soreness, elevated heart rate, or subjective fatigue—cannot by itself be taken as evidence that durable adaptation will follow; conversely, the absence of a pronounced acute response does not establish that consolidation has failed. Whether a given session’s adaptive information is ultimately consolidated depends on the subsequent biological processes addressed in Section 2.2, not on the magnitude of the initiating signal alone, a distinction with direct relevance to the common training practice of using acute session-level markers as a proxy for long-term adaptive progress.

Current exercise physiology models provide increasingly sophisticated descriptions of both the molecular signals activated during exercise and the structural adaptations that emerge following repeated training. However, considerably less attention has been devoted to the biological processes responsible for transforming transient adaptive information into durable physiological remodeling. Although the individual components of this transition, including protein synthesis, mitochondrial remodeling, immune regulation, extracellular matrix remodeling, and epigenetic modification, have been extensively investigated, they have rarely been considered within a unified conceptual framework. Therefore, understanding how exercise-induced adaptive information is transformed into durable physiological adaptations represents one of the remaining conceptual challenges in exercise physiology.

It is important to state precisely what is proposed as new here. The individual biological components synthesized in this review—signal transduction, transcriptional and translational regulation, protein turnover, chromatin remodeling, sleep-dependent recovery, and heritable variability in training response—are each well established in their own bodies of literature, and we do not claim otherwise. What has not, to our knowledge, been previously proposed is (i) the explicit organization of these components into a single ordered transition (signal generation, consolidation, gating, efficiency) that specifies testable boundary conditions at each step; (ii) the treatment of sleep not as one general recovery input among several, but as a domain-conditional gate whose strength is predicted to differ systematically across physiological systems, a claim that generates the specific, falsifiable predictions in Section 3; and (iii) the explicit reframing of interindividual variability determinants as components of a single efficiency construct (ACE) rather than as a list of independently studied factors. We do not claim that any single mechanistic finding cited in this review is new; the contribution is the integrative structure and the falsifiable predictions it generates, not the discovery of new biology.

### 2.2. Adaptive Consolidation

The evidence summarized above indicates that exercise-induced signaling alone cannot fully explain the emergence of durable physiological adaptations. Over the past decades, exercise physiology has made remarkable progress in characterizing the molecular pathways activated during and immediately after exercise, and recent advances in transcriptomics, epigenomics, proteomics, and multi-omics have considerably expanded our understanding of the biological complexity underlying exercise adaptation. Nevertheless, these advances have largely focused on either the initiation of adaptive signaling or the structural and functional outcomes observed after repeated training. Rather than reflecting a lack of mechanistic knowledge, this gap primarily arises from the absence of an integrative framework capable of organizing biological processes into a coherent model of exercise adaptation [1,19].

To address this conceptual gap, we propose Adaptive Consolidation as a systems-level framework describing the biological transition through which exercise-induced adaptive information is progressively stabilized and incorporated into durable physiological remodeling. Within the present framework, Adaptive Consolidation should not be interpreted as a novel molecular pathway or an independent physiological mechanism. Rather, it represents a conceptual organization of established biological processes that collectively determine whether transient exercise-induced responses ultimately become persistent.

Mechanistically, this transition can be described as a transduction cascade with a defined sequence: transient homeostatic perturbation, sensor activation, post-translational modification of downstream effectors, transcriptional and translational output, coordinated protein accumulation and degradation, and ultimately, structural and functional remodeling. Each step in this cascade constitutes a candidate site at which repeated exercise bouts are integrated into a stable physiological signature. The supporting evidence for this cascade spans human skeletal muscle studies and rodent or cell-based genetic models, where a specific mechanistic claim rests primarily on animal or cellular evidence, which is noted explicitly below, since the strength of causal inference differs considerably between genetic loss-of-function studies in mice and observational time-course studies in humans.

**Homeostatic perturbations are sensed by dedicated kinases.** Muscle contraction concurrently shifts intracellular calcium availability, energy charge (increasing AMP/ATP and ADP/ATP ratios), redox state, and mechanical tension. These variables are read by distinct sensor kinases: CaMKII for calcium, AMPK for energy charge, p38 MAPK for redox and stress signaling, and mTORC1 for mechanical and nutrient input. While this sensing architecture was long framed around a small set of kinases, recent phosphoproteomic analyses have indicated a substantially larger network of exercise-regulated kinases and substrates than previously appreciated [21].

Sensor kinases modify transcriptional effectors rather than directly building tissues. AMPK phosphorylates PGC-1α and Ulk1; calcium-mediated histone deacetylase efflux from the nucleus derepresses MEF2-dependent gene targets; and p38 MAPK phosphorylates PGC-1α and ATF2. This post-translational layer is illustrated by the proposed model of PGC-1α translocation following a single bout of exercise, in which PGC-1α is thought to relocate to both the nucleus and mitochondria, forming a complex with mitochondrial transcription factor A (TFAM) at the mitochondrial DNA D-loop and binding the nuclear respiratory factor 1 (NRF-1) promoter to coordinate nuclear and mitochondrial transcriptional programs. This model derives primarily from cellular and animal evidence; a re-evaluation of the corresponding human skeletal-muscle literature found that exercise-induced up-regulation of nuclear- and mitochondrial-encoded genes does not appear to occur in a clearly coordinated fashion in human skeletal muscle, indicating that this specific coordination step remains less firmly established in humans than the broader post-translational sensing mechanisms described above [22]. The overall regulatory logic is that mitochondrial biogenesis and the broader adaptive response are driven by the phosphorylation and translocation of nuclear and mitochondrial protein substrates rather than through sustained transcriptional activation alone [21].

**Transcriptional activation is transient and time-limited.** As described in Section 2.1, the induced transcriptional pulse is short, typically peaking between 1–4 h post-exercise and returning to baseline within roughly a day [3]. This transient transcriptional pulse constitutes the currency by which each exercise bout registers within the broader adaptive process.

**Protein accumulation operates as a low-pass filter for repeated transcriptional pulses.** Because individual mRNA pulses are short-lived, whereas many structural and enzymatic proteins have considerably longer half-lives, repeated transcriptional pulses are progressively integrated into monotonic protein-level accumulation. This is most clearly illustrated by the 14-day cycling time course described above, in which mitochondrial protein content rose steadily despite the underlying transcriptional response remaining transient after the first session [4].

**Coordinated degradation shapes the accumulation of specific proteins.** Adaptive Consolidation is not solely a synthetic process; the same transient signal that promotes biogenesis also flags components for removal. In a mouse model, a single bout of treadmill exercise induced transient mitochondrial oxidative stress within 3–12 h and mitophagy at approximately 6 h, preceded by AMPK-dependent phosphorylation of Ulk1 at Ser555. The genetic loss of Ulk1 abolishes exercise-induced metabolic adaptation, indicating that biogenesis alone is insufficient to produce the trained phenotype and that coordinated organelle turnover is required [23]. Consistent with this, PGC-1α appears to coordinate both biogenesis and mitophagy following acute exercise in animal and cell-based models, linking the synthetic and degradative arms to a common upstream signal [24]. Therefore, net structural remodeling can be understood as the difference between synthesis and degradation, both of which are triggered by the same initial transient perturbation.

**The chromatin state provides a durable memory linking successive exercise bouts.** Although transcription resolves within hours, the underlying chromatin accessibility landscape persists considerably longer and is progressively reshaped by training. Chromatin accessibility mapping performed immediately after exercise and at 6 and 72 h post-exercise showed that trained skeletal muscle exhibits more pronounced and more rapid chromatin closure immediately after exercise, with accessibility changes retained up to 72 h, alongside altered activation dynamics of immediate-early transcription factors such as Fos and Jun [25]. This provides a plausible mechanism by which the biological information carried by one exercise bout is retained until the next bout. The chromatin landscape encountered by a subsequent transient signal is not identical to that encountered by the first.

**Downstream effector programs are modality-specific.** In a rodent model, mTORC1 activity has been shown to be required for resistance-exercise-induced ribosome biogenesis and to contribute—though only partially, since mTOR-independent mechanisms also operate—to hypertrophy [26]. For endurance-type stimuli, the corresponding transductional arm converges on PGC-1α and the NRF/TFAM axis. In both cases, the same general transduction logic recruits a modality-specific transcriptional and translational program appropriate for the type of mechanical and metabolic perturbation imposed.

**Consolidation extends beyond the myofibers.** Durable adaptation also involves remodeling of the extracellular matrix, tendon mechanical properties, capillary density, satellite cell dynamics, and neural drive. This broader tissue-level remodeling has been described as a form of training-induced cellular memory, integrating neural adaptations, tendon and muscle fiber property changes, extracellular matrix remodeling, and altered inflammatory responses, and has been interpreted through a hormetic framework in which repeated sublethal stressors progressively activate adaptive gene programs that increase future physiological tolerance [27].

#### 2.2.1. Immune Remodeling as a Coordinated Consolidation Domain

The innate and adaptive immune systems display the same transient perturbation-then-integration architecture described above for the muscle transcriptional cascade, but are distributed across three temporally distinct waves. The fastest wave, leukocyte redistribution, unfolds within minutes: total leukocyte, lymphocyte, and neutrophil counts rise within five minutes of exercise onset, with most subsets peaking at 30–60 min into the bout and neutrophils peaking later, at approximately 3 h post-exercise, reflecting an initial catecholamine-driven demargination followed by a delayed, cortisol-driven bone-marrow release [28]. This redistribution rapidly reverses upon cessation of exercise, with most lymphocyte and monocyte subsets falling below their end-exercise values within three minutes of recovery, and total leukocyte counts returning to baseline within approximately a day. Second, a cytokine-centered wave follows a slower but still self-limiting course: plasma interleukin-6 rises during exercise and peaks at the end of the bout, with interleukin-1 receptor antagonist peaking somewhat later, during the first hours of recovery; this cytokine wave typically returns to near-baseline within 24–48 h, even after marathon-distance efforts, in a pattern that scales with exercise dose [29,30]. The slowest wave, the hepatic acute-phase response (C-reactive protein, fibrinogen), lags the cytokine signal by design, rising over the first 24–48 h post-exercise and returning to baseline over the following two to seven days, with the recovery time scaling with the magnitude and duration of the exercise stimulus [30,31]. This three-wave architecture is structurally analogous to the muscle transcription cascade described above—transient perturbation followed by a return to baseline—but uses circulating immune cells and hepatic proteins rather than muscle mRNA as the readout.

Beyond this repeatedly resolving acute pattern, a growing body of evidence indicates that repeated exercise leaves a durable structural residue in the immune system, functioning as a low-pass filter, analogous to the protein-level accumulation described in Section 2.2 for skeletal muscle. In a mouse model, voluntary exercise reduced hematopoietic activity and altered the epigenome and transcriptome of bone marrow hematopoietic progenitor cells; critically, while the upstream hormonal trigger (reduced leptin) was quickly reversed upon exercise cessation, the resulting changes in leukocyte production and hematopoietic progenitor epigenome persisted for several weeks after training was withdrawn—an explicit dissociation between a transient upstream signal and a persistent structural residue [32].

In humans, this bone marrow-level reprogramming appears to manifest peripherally as a stable, between-bout reduction in pro-inflammatory CD14^+^CD16^+^ monocytes and their lipopolysaccharide-stimulated cytokine output following 12 weeks of endurance or resistance training in previously sedentary adults [33,34]. At the tissue level, endurance training increases the accumulation of anti-inflammatory M2-polarized macrophages in human skeletal muscle, which correlates with fiber hypertrophy and satellite cell content [35]. The Treg cell mechanism appears to be causally, not merely correlationally, required for the muscle metabolic benefits of exercise. In a mouse model, exercise rapidly expands the muscle-resident Treg cell compartment, which limits interferon-γ-mediated mitochondrial damage, and genetic ablation of these cells blunts exercise-induced gains in exercise capacity [36]. At the population level, this cumulative immune remodeling is reflected in a modest but consistent reduction in resting C-reactive protein levels with exercise training across meta-analytic and large cohort data, an effect that is most pronounced in individuals with elevated baseline inflammation [37,38].

Taken together, the immune arm of Adaptive Consolidation shows the same defining features already established for the muscle-transcriptional and mitochondrial arms: a long-lived cellular substrate that outlives any single bout (bone marrow progenitors, tissue-resident macrophages, and regulatory T cells) and at least one instance of demonstrated causal necessity for the downstream physiological adaptation. An important caveat, however, is that the durability of this remodeling in humans after training cessation is considerably less well characterized than in the rodent hematopoietic-progenitor model, where the “several weeks” persistence estimate currently provides the best available benchmark [32].

#### 2.2.2. Autonomic Regulation as a Durable Consolidation Output

Resting cardiac autonomic tone, most commonly indexed by heart-rate variability (HRV) and baroreflex sensitivity, satisfies the same criteria used elsewhere in this section to identify a genuine consolidation output: a quantifiable, dose-dependent change following repeated training, an identifiable durable substrate, and reversibility upon withdrawal of the stimulus. Meta-analytic evidence across randomized controlled trials indicates that exercise training reliably raises resting vagal-related HRV indices—including SDNN, RMSSD, and high-frequency power—relative to non-training controls, with the effect present in previously sedentary adults and persisting into older age, where it scales linearly with the training frequency [39].

This is not solely a change in autonomic tone acting on an unchanged heart; in previously sedentary older adults, six months of aerobic training increased arterial baroreflex gain by approximately one quarter, an increase attributable predominantly to the neural component of the reflex arc rather than to arterial mechanical properties [40], while training-induced resting bradycardia in animal and human studies is partly explained by a genuine reduction in intrinsic (non-neurally mediated) heart rate, accompanied in elite human cyclists by structural left-ventricular remodeling that correlates inversely with intrinsic heart rate [41,42].

In a rodent hypertension model, training-induced restoration of parasympathetic and baroreflex function was accompanied by measurable neuroplastic changes within the central autonomic nuclei of the hypothalamus and brainstem, indicating that this consolidation domain, like the muscle-chromatin memory described earlier in this section, has an identifiable durable substrate, although in this case within the central nervous system rather than in muscle [43]. This trained autonomic phenotype behaves as a reversible adaptation rather than a fixed trait; detraining studies show that training-elevated resting HRV and accelerated post-exercise vagal reactivation revert toward pre-training baseline over a comparable multi-week timescale to that over which they were acquired [44,45]. Because the same physiological signal is used at three different timescales within this review—nocturnal, sleep stage-locked HRV as a mechanistic correlate of Sleep-Gated Adaptation (Section 2.3), the resting trained baseline shift in HRV and baroreflex sensitivity described here as a consolidation output, and morning HRV as a monitoring biomarker (to be addressed in future work)—these three readings should be understood as complementary resolutions of one underlying regulatory system rather than as competing definitions.

Taken together, these observations indicate that Adaptive Consolidation is not reducible to a single mechanism but rather emerges from the composition of multiple distinguishable regulatory filters and domains—post-translational modification, transcription, translation, coordinated degradation, chromatin remodeling, immune-cell reprogramming, and autonomic remodeling—each operating on a different timescale and representing a point at which the biological information generated by exercise may be reinforced, attenuated, or resolved. Current evidence consistently indicates that successful adaptation depends on the coordinated interaction of these processes rather than the isolated activation of any single signaling pathway, and that repeated exercise induces coordinated remodeling across multiple tissues rather than sustained activation of isolated pathways [19,46].

From this perspective, physiological adaptation should be regarded as a progressive biological process rather than a direct consequence of acute molecular activation. Exercise initiates adaptive signaling within minutes, whereas the structural and functional remodeling responsible for long-term adaptation develops over hours, days, and repeated training cycles. During this interval, biological responses are continuously integrated, amplified, attenuated, or resolved before stable physiological adaptations are established. Consequently, the generation of adaptive signals represents only the first stage of the adaptive process; the biological events occurring throughout the postexercise period appear to play an equally important role in determining whether exercise-induced adaptive information is successfully translated into durable physiological remodeling [1,3,19].

Therefore, Adaptive Consolidation does not replace the current molecular models of exercise adaptation. Instead, it provides a conceptual framework through which existing biological knowledge can be interpreted from a common systems perspective. By explicitly recognizing the intermediate biological phase linking exercise-induced signaling with long-term physiological remodeling, this framework integrates processes that have traditionally been investigated separately into a unified interpretation of exercise adaptation. In this sense, Adaptive Consolidation serves primarily as an organizing principle that connects established mechanisms rather than introducing new biological mechanisms.

This perspective raises an important biological question. If durable physiological adaptation depends on the coordinated interaction of multiple biological systems during the postexercise period, which physiological state provides the most favorable conditions for this integration to occur? Addressing this question forms the basis of the Sleep-Gated Adaptation (SGA) framework proposed in the following section.

### 2.3. The Sleep-Gated Adaptation Framework

The concept of Adaptive Consolidation provides a systems-level perspective for understanding how exercise-induced adaptive information may be progressively translated into durable physiological remodeling. However, this framework also raises a fundamental biological question: under which physiological conditions can the coordinated integration of these adaptive processes occur most effectively?

Sleep is a unique physiological state characterized by the simultaneous coordination of multiple biological systems that are individually recognized as important regulators of exercise adaptation. Endocrine regulation, autonomic recalibration, immune modulation, protein turnover, mitochondrial maintenance, metabolic restoration, neural plasticity, and circadian organization undergo profound and highly coordinated changes during sleep. Although these processes have traditionally been investigated independently, the convergent human evidence summarized below indicates that sleep provides a distinctive biological environment capable of supporting the integration of adaptive responses initiated during the wakefulness.

Within this biological context, we propose the Sleep-Gated Adaptation (SGA) framework. SGA describes the principle that a biologically permissive sleep state shapes how efficiently exercise-induced adaptive information is consolidated into durable physiological adaptations, without implying that such a state is strictly necessary for any consolidation to occur. Importantly, this framework does not imply that sleep directly generates such adaptations. Exercise remains the primary stimulus responsible for initiating adaptive signaling, whereas sleep provides the coordinated physiological conditions under which information can be integrated across multiple biological systems. Accordingly, SGA should be interpreted as a biological gating principle, rather than an independent adaptive mechanism.

Several convergent lines of human evidence support the biological plausibility of a sleep-dependent consolidation gate, although the strength of this dependency differs considerably across the adaptive arms.

**Anabolic hormonal window.** The largest daily pulse of growth hormone in men is coupled to sleep onset and slow-wave sleep, with a near-linear relationship between the amount of slow-wave sleep and concurrent growth hormone secretion; both decline exponentially with age [5]. This places a major anabolic signal—one that would ordinarily amplify post-exercise IGF-1 signaling and protein synthesis—inside a specific sleep architecture rather than merely within a period of rest. Even a single night of total sleep deprivation is sufficient to disrupt this hormonal environment, producing a 21% increase in plasma cortisol and a 22% decrease in testosterone [6].

**Muscle protein synthesis.** This is the most direct sleep-dependent adaptive arm. In the same acute deprivation paradigm, the muscle fractional synthesis rate fell by 18% after a single night without sleep, a pattern the original authors described as a state of anabolic resistance [6]. Sustained sleep restriction produces a comparable effect: five nights of 4-h sleep reduced myofibrillar protein synthesis in healthy young men [7]. Notably, this deficit was not absolute; three sessions of high-intensity interval exercise performed during the same sleep-restricted week restored myofibrillar protein synthesis to control levels [7], indicating that exercise itself can partially compensate for the reduced sleep-dependent anabolic drive. The endocrinological framework proposed to explain this pattern—sleep debt raising cortisol while lowering testosterone and IGF-1, shifting the systemic balance from protein-synthetic to proteolytic signaling—remains consistent with subsequent findings [8]. This five-night sleep-restriction design, with and without concurrent high-intensity exercise, constitutes the closest available empirical analog in this literature to a graded manipulation of sleep-dependent consolidation, since it varies both the severity of the sleep insult and the presence of a compensatory exercise stimulus within the same protocol.

**Mitochondrial adaptation and metabolic control.** A companion investigation to the myofibrillar synthesis study provides the clearest demonstration that mitochondrial adaptation is sleep-dependent: five nights of sleep restriction reduced mitochondrial respiratory function, sarcoplasmic protein synthesis, glucose tolerance, and the amplitude of the diurnal skin temperature rhythm; critically, adding three high-intensity interval exercise sessions to the same sleep-restricted week prevented all of these deficits [9]. This indicates that the efficiency with which the transient AMPK–PGC-1α signaling described in Section 2.2 is translated into mitochondrial-level adaptation depends on a permissive sleep environment, while also confirming that exercise can partially rescue the sleep-dependent deficit rather than consolidation being wholly dependent on sleep.

**Circadian alignment as a co-regulator.** The influence of sleep loss is not limited to endocrine signaling; skeletal muscle metabolism operates under robust circadian control, with core clock genes contributing to muscle development and function, and the muscle molecular clock is increasingly framed as a co-regulator of the exercise-induced transcriptional response [47]. As sleep restriction blunts the amplitude of peripheral diurnal rhythms [9], disrupted sleep may desynchronize this clock, such that the next acute exercise-induced transient arrives at a chromatin and transcriptional state that is not optimally primed to receive it.

**Motor memory consolidation.** Skill-related adaptation—technique and coordination—shows an explicit sleep-dependent step with no demonstrated waking equivalent. Following training on a motor-sequence task, retesting 12 h later after an intervening sleep period, rather than an equivalent waking period, was associated with measurable functional reorganization of the motor representation, including increased activation in the primary motor cortex, cerebellum, and hippocampus, alongside reciprocal decreases in the parietal and insular regions, supporting faster and more precise movement execution [10]. This suggests that the neural component of exercise-induced adaptation may be more strictly sleep-gated than the mitochondrial or protein-synthetic components of exercise-induced adaptation.

**A bidirectional relationship exists between injuries and sleep.** Sleep is not only an input for post-exercise recovery; tissue damage appears to drive subsequent sleep. Experimentally induced muscle injury in an animal model produced a significant increase in total sleep time and specifically non-rapid eye movement sleep at 48–72 h post-injury, associated with decreased brain IGF-1 and altered muscle *BMAL1* expression [48]. This finding is consistent with sleep functioning as an active, demand-responsive phase of the repair program, rather than a passive background state.

**The post-exercise transcriptomic program is reshaped, not merely delayed, under sleep debt.** The most direct test of whether sleep restriction alters the adaptive response itself, rather than only its magnitude, comes from a resistance exercise paradigm in young females: nine nights of sleep restriction combined with four resistance exercise sessions produced roughly 3000 differentially regulated transcripts 48 h post-exercise under both sleep conditions, but only 39% of downregulated and 18% of upregulated genes overlapped between sleep-restricted and normal sleep conditions [49]. The authors concluded that performing exercise under sleep restriction may not elicit the same adaptive response as the same exercise performed while fully rested [49]—direct evidence that inadequate sleep changes the qualitative content of the adaptive signal, not merely its quantitative resolution.

**Damage recovery**. In an animal model, 72 h of sleep deprivation elevated post-exercise markers of muscle damage and reduced recovery capacity, an effect attributed to reduced protein synthesis under sleep-restricted conditions [50].

**The postexercise immune response is conditionally, rather than uniformly, sleep-dependent.** Human experimental evidence on whether sleep restriction alters the post-exercise immune waves described in Section 2.2.1 is genuinely mixed, and the pattern of when an interaction is observed is itself informative. Positive findings were consistently associated with three co-occurring conditions: a damaging or prolonged exercise stimulus, a total or multi-night sleep insult, and a delayed readout window. Under these conditions, partial sleep deprivation has been shown to amplify exercise-induced interleukin-6 and tumor necrosis factor-α responses in team-sport athletes [51]; combining a standardized eccentric-damage protocol with 48 h of total sleep deprivation selectively elevates interleukin-6 relative to a matched normal-sleep condition, despite comparable strength recovery [52]; competitive rugby league match play followed by a fully sleep-deprived night produces larger creatine-kinase and C-reactive-protein responses at 16 h post-match than an equivalent night of normal sleep [53]; and prior sleep disruption augments the magnitude of exercise-induced lymphocyte redeployment despite matched cortisol and catecholamine responses [54]. Conversely, studies combining a brief or single-night sleep insult with short-duration, non-damaging aerobic exercise, and an early post-exercise readout have consistently failed to detect an interaction, including the most methodologically rigorous test to date—a five-night, 4-h sleep-restriction protocol with concurrent high-intensity interval exercise—which found no change in plasma interleukin-6, tumor necrosis factor-α, or IFN-γ, with the sole exception of an increase in skeletal-muscle NFAT1 protein [55]. This boundary condition pattern indicates that the immune arm of Adaptive Consolidation is sleep-gated specifically in damage-repair and cumulative-load contexts rather than universally, and that null findings from short-insult, low-damage designs should not be read as evidence against sleep dependence in the contexts most relevant to training practice.

**Sleep stage-locked autonomic recovery provides the most direct mechanistic evidence for a permissive sleep gate.** Cardiac parasympathetic tone, indexed by heart-rate variability, is not a passive overnight average but tracks sleep stage in real time; parasympathetic activity is highest during slow-wave sleep and is measurably attenuated during rapid-eye-movement sleep, a relationship that extends to functional connectivity within central autonomic brain regions during slow-wave sleep [56,57]. Critically, this relationship has been shown to be causal rather than merely correlational: experimentally enhancing slow-wave activity with closed-loop acoustic stimulation increases parasympathetic heart-rate-variability power and reduces overnight sympathetic drive, establishing that the direction of causation runs from sleep architecture to the autonomic state [58].

Mechanistically, this state-dependence is thought to reflect the coordinated withdrawal of sympathetic outflow and the reciprocal dominance of cardiac parasympathetic tone that characterizes slow-wave sleep, a pattern that has been linked to coordinated activity within central autonomic brain regions during this sleep stage specifically [57]. This provides one of the more concrete candidate mechanisms in this review linking a specific sleep stage to a specific, measurable physiological process, in contrast to the less mechanistically resolved endocrine and immune domains discussed above, where the sleep-dependent step is evidenced primarily through outcome measures (hormone concentration, cytokine level) rather than through an identified intermediate physiological pathway.

This mechanism appears to be the physiological substrate for postexercise autonomic recovery. Specifically, vagal-related heart-rate-variability indices measured during slow-wave sleep are measurably depressed on the night following intense exercise and recover over the subsequent nights [59]. Slow-wave sleep variability discriminates training-load state more sensitively than waking measurements [60], and functionally overreached endurance athletes show a selective reduction in parasympathetic control specifically during slow-wave sleep without a corresponding change across the whole-night average, a deficit that resolves with tapering [61]. A systematic review of postexercise cardiac parasympathetic reactivation places full recovery at up to 24 h after low-intensity exercise, 24–48 h after threshold-intensity exercise, and at least 48 h after high-intensity exercise [62]. Sleep restriction disrupts this mechanism directly rather than only its downstream readout: polysomnographically confirmed partial sleep restriction increases sympathetic tone and decreases vagal tone during the sleep period itself, not merely upon waking [63].

The clearest experimental evidence for a sleep–exercise–autonomic interaction is confined to short recovery windows: partial sleep restriction has been shown to blunt vagal-related heart-rate-variability indices in the minutes following exercise and to reduce heart-rate recovery specifically in the first 30–60 s after a maximal effort in both adolescent and adult cohorts, with the deficit consistently localized to the earliest reactivation phase rather than distributed evenly across several minutes [64,65]. A functional analog directly comparable to the transcriptomic evidence discussed above showed that partial sleep deprivation impairs 24-h recovery of peak power output following a single training session [66]. However, the best-controlled test of the intermediate, multi-hour post-exercise heart-rate-variability recovery curve found that while high-intensity interval exercise depressed heart-rate variability for up to 4 h post-exercise, acute partial sleep deprivation did not further modify that recovery curve [67]—an honest null that constrains the claim to the earliest reactivation phase and the overnight window, rather than the entire multi-hour recovery period.

Taken together, the evidence summarized above—now extended to the immune and autonomic domains—indicates that sleep-dependent adaptive arms differ substantially in the strength and conditionality of their dependency. Protein-synthetic anabolism (growth hormone–testosterone–IGF-1 axis, myofibrillar and sarcoplasmic synthesis), motor-memory consolidation, and the earliest phase of post-exercise parasympathetic reactivation, for which mechanistic and, in the case of slow-wave sleep manipulation, causal evidence exists, show the strongest evidence of sleep dependency. Mitochondrial respiratory adaptation, circadian amplitude and clock alignment, and the qualitative shape of the post-exercise transcriptomic program show consistent but less direct mechanistic evidence. The post-exercise immune response and the intermediate, multi-hour phase of autonomic recovery show the most conditional pattern: sleep dependence is detectable under damaging or prolonged exercise combined with sustained sleep loss and delayed readouts, but is not reliably detectable with brief sleep insults, non-damaging exercise, or early window readouts alone [6,7,9,10,49,51,52,53,54,55,56,57,58,59,60,61,62,63,64,65,66,67].

Table 1 arranges the eight consolidation domains introduced in Figure 1 along this evidence gradient, from strongest to most conditional, so that the domain-specific pattern established above can be consulted directly rather than reconstructed from the preceding narrative.

The tiers in Table 1 were assigned using three criteria applied consistently across domains: (i) whether at least one causal or interventional manipulation (as opposed to purely observational association) was available; (ii) whether the finding had been reported in more than one independent human study; and (iii) whether a plausible mechanistic pathway connecting sleep state to the outcome had been proposed. A domain was assigned to the ‘strongest’ tier only if criteria (i) and (ii) were both met; to ‘consistent’ if criterion (iii) was met together with either (i) or (ii) but not both; and to ‘conditional’ if the interaction was reported in only a single study, was reported alongside a credible null finding, or was detectable only under a narrow combination of boundary conditions. We emphasize that these criteria are considerably less formal than standard evidence-grading systems such as GRADE, do not incorporate a structured risk-of-bias assessment of individual studies, and should not be interpreted as equivalent to a systematic evidence synthesis; they are reported here only so that the basis for the tier assignments in Table 1 is transparent and checkable rather than implicit.

**A necessary refinement of the Sleep-Gated Adaptation framework.** An important nuance qualifies SGA as proposed here: exercise itself appears to partially rescue several sleep-dependent adaptive arms, even under conditions of sustained sleep restriction. High-intensity interval exercise preserved myofibrillar protein synthesis [7] and mitochondrial respiratory function [9] during a five-night restriction protocol that otherwise produced clear deficits, and habitual high-intensity physical activity also attenuates the impact of acute total sleep deprivation on heart rate variability [68]. Therefore, the SGA should not be interpreted as a strict, all-or-none biological switch in which adaptation is blocked in the absence of adequate sleep, nor as a uniformly acting gate across every adaptive arm and recovery window. Rather, the available evidence supports a graded, permissive, and domain-conditional gating principle: sleep loss shifts the balance of synthesis versus degradation, chromatin accessibility, neural consolidation, immune signaling, and early phase autonomic reactivation, but the magnitude and even the detectability of this shift depend on the severity and duration of the sleep insult, the damage load and duration of the preceding exercise bout, and the specific recovery window under examination, and the shift can be partially, though evidently not fully, offset by the exercise stimulus itself.

Therefore, the proposed framework distinguishes between two complementary stages of the adaptive process. The first stage involves the generation of adaptive information through exercise-induced signaling. The second stage involves the biological consolidation of this information during the postexercise period, a process proposed to be regulated by sleep-dependent physiological coordination. This distinction provides a conceptual explanation for why comparable exercise stimuli may result in markedly different long-term adaptations, despite broadly similar acute molecular responses. However, as the evidence on interindividual variability discussed in Section 2.4 demonstrates, sleep-dependent gating is only one of several determinants of this outcome.

While Sleep-Gated Adaptation describes the biological principle governing adaptive consolidation, it does not describe the effectiveness with which consolidation occurs. Therefore, we introduce Adaptive Consolidation Efficiency (ACE) as a functional systems-level construct describing how efficiently exercise-induced adaptive information is ultimately translated into durable physiological adaptation. ACE is not intended to be a biomarker, physiological index, or quantitative score. Rather, it represents an emergent biological property that reflects the overall effectiveness of adaptive consolidation across multiple interacting physiological systems, the empirical determinants of which are examined in the following section.

Within the proposed hierarchy, the SGA and ACE perform distinct but complementary functions. Sleep-Gated Adaptation explains why adaptive consolidation succeeds or fails by defining the biological conditions under which consolidation becomes possible. Adaptive Consolidation Efficiency explains how effectively consolidation ultimately occurs, given a permissive sleep state. Together, these complementary concepts provide a unified framework linking exercise-induced signaling, sleep physiology, and long-term adaptations (Figure 1 and Box 1).

Box 1 **The SGA–ACE Hierarchy.**

**1. Exercise generates adaptive signal.**

Exercise initiates an adaptive process by generating exercise-induced adaptive information through coordinated molecular and physiological signaling. This information alone does not constitute durable adaptation; it must undergo a subsequent phase of biological integration, referred to as Adaptive Consolidation (Section 2.2).

**2. Sleep shapes how efficiently these signals can be biologically consolidated through Sleep-Gated Adaptation (SGA).**

The SGA describes the biological principle that sleep provides the most favorable physiological conditions for consolidation by coordinating multiple adaptive systems during the postexercise period, functioning as a graded rather than strictly binary gate (Section 2.3).

**3. Adaptive Consolidation Efficiency determines how efficiently adaptive information becomes a durable physiological adaptation (ACE).**

ACE describes the functional, individual-specific consequence of this biological gate: the overall effectiveness with which exercise-induced adaptive information is ultimately translated into durable physiological adaptation, as modulated by the determinants examined in Section 2.4.

**→Long-Term Physiological Adaptation**

### 2.4. Adaptive Consolidation Efficiency

The Sleep-Gated Adaptation framework accounts for the physiological conditions under which consolidation becomes possible, but it does not, by itself, explain why individuals exposed to comparable exercise stimuli and sleep conditions still exhibit markedly different long-term adaptive outcomes. Interindividual variability in exercise adaptation is among the most extensively studied and contested phenomena in exercise physiology, and it provides the empirical foundation for Adaptive Consolidation Efficiency (ACE) as introduced in Section 2.3. Rather than proposing ACE as an abstract placeholder, this section examines the principal sources of variance identified in the literature and organizes them as ACE candidate determinants.

**Heritable variance in trainability**. The foundational evidence for a genetic contribution to adaptive outcomes comes from the HERITAGE Family Study, in which 481 sedentary adults from 98 families completed an identical 20-week endurance training program. The mean ΔVO_2_max was approximately 400 mL/min, but individual responses ranged from essentially no improvement to gains exceeding 1 L/min; the variance between families was 2.5 times greater than the variance within families, yielding a heritability of trainability of 47% [11]. A subsequent genome-wide association analysis identified 21 single-nucleotide polymorphisms that together explained 49% of the variance in ΔVO_2_max: individuals carrying nine or fewer favorable alleles gained on average 221 mL/min, whereas those carrying 19 or more favorable alleles gained 604 mL/min; the strongest single locus, *ACSL1*, accounted for approximately 6% of the variance on its own [12].

**Response is trait-specific rather than reflecting a global “responder type”.** This qualification substantially reframes the heritability estimates. A reanalysis of 566 HERITAGE participants across seven cardiometabolic traits (VO_2_max, body fat, resting heart rate, insulin, HDL cholesterol, small LDL particles, and glycoprotein acetylation) found only one participant who was a universal high responder and one who was a universal low responder; 51% of participants were simultaneously high responders on at least one trait and low responders on another, and correlations between different response traits ranged from −0.22 to 0.11 [13]. The clinical implication is that regular exercise is likely to produce multiple health benefits, even when a specifically targeted variable fails to improve [13]. This pattern is corroborated by evidence that individuals classified as VO_2_max “non-responders” nonetheless show improvements in cycling efficiency and other metabolic parameters [14].

**Low response is frequently modality-specific rather than intrinsic in nature.** A crossover twin study assigned 68 twin pairs to three months of endurance training, followed by three months of resistance training. Individual responses to the two modalities were uncorrelated, and approximately 50% of participants showed discordant responses—a poor response to one modality alongside a positive response to the other [17]. This has practical implications for the interpretation of apparent non-response: switching training modality can “rescue” many individuals previously classified as non-responders, indicating that part of what appears to be an intrinsic biological ceiling instead reflects a mismatch between individual biology and a specific training prescription [17].

**The genetic contribution to variability may be smaller than that suggested by early heritability estimates.** This twin study included both monozygotic and dizygotic pairs. Significant intraclass correlations for strength and cardiorespiratory fitness changes were observed in monozygotic but not dizygotic pairs; however, the monozygotic intraclass correlations were not statistically larger than the dizygotic correlations, which is the pattern that a genuine genetic signal is expected to produce. On this basis, the authors concluded that environmental factors, rather than genetics, account for most of the variance in strength and cardiorespiratory fitness responses to training [17]. This finding stands in some tension with the 47% heritability figure derived from HERITAGE; a plausible reconciliation is that the family-based HERITAGE design captured the shared environment in addition to the shared genotype.

**Epigenetic modifications as proximate mediators.** DNA methylation provides a plausible molecular bridge between the genotype, environment, and adaptive phenotype. Among lean sedentary men, high responders to acute exercise, defined by hypomethylation at a regulatory site in the *PGC-1α* promoter, showed a significant increase in *PGC-1α* expression and a trend toward decreased intramyocellular lipid content, whereas low responders did not [15]. Independently, long-term endurance training has been shown to change the DNA methylation pattern of human skeletal muscles, with training responsiveness related to differential gene expression through mechanisms that remain incompletely understood [16].

This epigenetic finding illustrates a gene–environment interaction directly relevant to ACE: the same exercise stimulus (environment) produces divergent molecular outcomes depending on pre-existing methylation status at the *PGC-1α* promoter (a genetically and developmentally influenced trait), such that a given training prescription cannot be assumed to act on a biologically uniform substrate across individuals.

**Baseline fitness shapes both the magnitude and the apparent rate of response.** Evidence from a standardized training cohort indicates that within groups, less-fit individuals tend to show larger absolute improvements, whereas between groups, fitter individuals respond at a faster relative rate to an identical training stimulus, even after the less-fit group receives additional preparatory training [69]. This absolute-versus-relative distinction is relevant to the ACE: greater room for improvement plausibly explains the within-group pattern, while a biological ceiling effect may explain the between-group pattern. This finding is reported in a conference abstract rather than a full peer-reviewed article [69] and should be weighted accordingly relative to the full-text sources cited elsewhere in this section.

**Biological sex contributes to hormonally mediated sources of variance.** Transcriptomic profiling of resting skeletal muscle indicates that sex differences are largely explained by circulating testosterone and estradiol rather than by Y-chromosome gene expression, and that men and women show sex-dependent transcriptomic responses to aerobic, resistance, and combined training [70]. Because these hormonal profiles shift with age, this factor plausibly interacts with age-related variability. Together with the modality-mismatch findings discussed above, this hormonal evidence illustrates the same gene–environment logic applied to a different determinant: an individual’s hormonal milieu (an endogenous, partly genetically influenced factor) interacts with the specific exercise modality prescribed (the environmental exposure) to jointly shape the adaptive outcome, rather than either factor acting independently.

**Aging blunts and diversifies the adaptive response.** Older adults show blunted and more variable responses across several adaptive systems, including protein synthesis, mitochondrial biogenesis, and the ceiling for VO_2_max improvements. A recent workshop synthesis concluded that adequately powered prospective studies in adults over 70 years of age remain overdue, and that both intrinsic factors (genetic and epigenetic) and extrinsic factors (medication use, diet, and chronic disease burden) require systematic investigation as determinants of adaptive capacity in this population [71].

**Environmental and lifestyle modifiers converge directly with the Sleep-Gated Adaptation framework.** As detailed in Section 2.3, sustained sleep restriction reshapes the qualitative content of the postexercise transcriptomic program, with only 18–39% overlap in exercise-regulated genes between rested and sleep-restricted conditions [49]. Within the present framework, this evidence should be interpreted as a direct environmental determinant of ACE rather than solely as evidence for the SGA gate: sleep does not only determine whether consolidation can occur, but also contributes to the amount of adaptive information generated by a given bout that is ultimately retained. Other environmental factors, including nutrition, medication use, and the gut microbiome, may act through comparable pathways. Endurance training has been associated with shifts toward lactate-metabolizing and propionate-producing microbial taxa, with microbial metabolites proposed to influence energy regulation, immune function, and recovery, although causal evidence in this area remains limited [72].

**Training prescriptions are a source of apparent, but not necessarily intrinsic, non-response.** As noted above, modality mismatch accounts for a substantial share of individuals who appear to be biological non-responders when tested using a single training mode [17]. This implies that ACE should not be modeled purely as an individual trait but as the outcome of an interaction between individual biology and the specific characteristics of the applied exercise stimulus.

**A portion of the observed variability reflects measurement errors rather than biology.** A methodologically important caveat qualifies the entire variability literature: many individuals classified as “low responders” based on a single training block are not reproducibly classified as low responders when the identical block is repeated, indicating that within-subject measurement variability inflates the apparent magnitude of interindividual variability [18]. Consequently, some portion of what is described in the literature as biological non-response is statistical noise rather than a stable individual characteristic, a caveat that should temper any attempt to operationalize ACE as a fixed, trait-like quantity.

This distinction—between observed variability in a single measurement and true, stable interindividual heterogeneity once within-subject noise, regression to the mean, and protocol-specific outcome selection are accounted for—is fundamental rather than a minor caveat, and it is not fully resolved by the literature synthesized in this section. Several of the foundational trainability estimates discussed above, including the HERITAGE heritability figure, derive from single-occasion outcome measurements and predate the more recent replicate-testing literature demonstrating this inflation. Consequently, some fraction of what is described throughout this section, and in the exercise physiology literature more broadly, as stable biological response heterogeneity may instead reflect measurement error, regression to the mean, or the specific outcome measure chosen, rather than a fixed individual trait. We do not consider this issue resolved, and it directly motivates the requirement in Section 3.5 that ACE-testing designs include repeated-measures or replicate testing sufficient to distinguish genuine low response from measurement noise, rather than relying on single-occasion classification as much of the cited literature has done.

**Synthesis: ACE as a multiply determined construct.** Taken together, the evidence reviewed in this section indicates that the efficiency with which exercise-induced adaptive information is translated into durable physiological adaptation is not attributable to a single dominant factor, but is distributed across at least six partially overlapping domains: heritable genetic variation, epigenetic state, baseline biological characteristics (fitness, sex hormone milieu, age), environmental and lifestyle modifiers (including sleep, as formalized in the SGA framework), the degree of match between individual biology and training prescription, and residual measurement variability (Box 2). Within the present framework, Adaptive Consolidation Efficiency is proposed as a system-level construct that conceptually integrates these sources of variance. Consistent with the definition introduced in Section 2.3, ACE is not proposed here as a measurable index, composite score, or biomarker; rather, it provides an organizing label for a well-documented empirical phenomenon—interindividual variability in exercise adaptation—that has previously been described piecemeal across genetic, epigenetic, endocrine, and environmental literature without a unifying systems-level term. A practically important corollary, drawn directly from the trait-specific reanalysis of the HERITAGE cohort, is that universal non-responders are essentially non-existent: an individual who fails to improve on one clinically relevant trait is very likely to improve on another [13]. Accordingly, low ACE should be understood as an outcome- and context-specific measure rather than a fixed individual property.

We acknowledge a specific circularity risk in the ACE construct as defined: if ACE is inferred only from the training outcome it is meant to explain, the construct adds a label rather than explanatory content. We attempt to constrain this risk in two ways, both of which are testable rather than assumed. First, the determinants proposed above (genetic variants, baseline methylation status, baseline fitness and hormonal profile, and prescription match) are, in principle, measurable prior to and independently of the training outcome itself, and prediction P6 (Section 3.2) explicitly requires that these determinants be measured at baseline and shown to explain outcome variance jointly, rather than being inferred post hoc from the outcome. Second, the falsification criterion attached to P6 is designed specifically to test whether ACE has independent content: if sleep-restriction exposure alone accounts for the substantial majority of outcome variance once measured, with the remaining determinants adding negligible independent explanatory power, this would indicate that the multi-determinant ACE construct is largely redundant with SGA and does not provide meaningful explanatory value beyond it (Section 3.6). We consider this a genuine, currently unresolved possibility rather than a remote alternative, and we present ACE as a hypothesis to be tested against this falsification criterion rather than as an established source of independent explanatory power.

Box 2 **Candidate Determinants of Adaptive Consolidation Efficiency (ACE).**

**Genetic**—heritability of trainability (≈47%, HERITAGE); polygenic contribution of ≈21 SNPs (≈49% of ΔVO_2_max variance); largest single locus *ACSL1* (≈6%) [11,12].

**Epigenetic**—exercise- and training-induced DNA methylation changes at loci such as *PGC-1α* differentiate responders from non-responders [15,16].

**Baseline biology**: Initial fitness level, sex hormone milieu, and age jointly shift the amplitude and kinetics of the adaptive response [69,70,71].

**Environmental/lifestyle factors**, such as sleep quantity and quality (Section 2.3), nutrition, and the gut microbiome, modulate the substrate on which the exercise signal acts [49,72].

**Prescription match**—modality specificity: an individual may be well-tuned to one training mode and poorly tuned to another, producing an apparent non-response that resolves with a modality switch [17].

**Measurement noise**: within-subject variability and imprecise outcome measures inflate the apparent magnitude of true biological non-response [18].

### 2.5. Synthesis of the Conceptual Framework

The framework developed in this section can be summarized in three sequential propositions. First, exercise generates adaptive signals: transient, multimodal molecular perturbations that encode—but do not themselves constitute—the information required for physiological adaptation (Section 2.1). Second, this information undergoes Adaptive Consolidation, a multi-filter transduction process spanning post-translational modification, transcription, protein turnover, and chromatin remodeling, through which repeated transient signals are progressively integrated into stable biological change (Section 2.2). Third, the extent to which this consolidation process succeeds is jointly governed by a permissive physiological gate—Sleep-Gated Adaptation—and by a distribution of individual-specific determinants captured under the label Adaptive Consolidation Efficiency, spanning genetic, epigenetic, baseline-biological, environmental, and prescription-related sources of variance (Section 2.3 and Section 2.4).

Consistent with the conceptual status of Adaptive Consolidation introduced in Section 2.2, neither SGA nor ACE is proposed as an established mechanism, a validated biomarker, or a quantitative index at this stage. Both terms function as organizing constructs intended to integrate mechanistically heterogeneous but empirically convergent bodies of literature—exercise molecular signaling, sleep physiology, and interindividual variability research—into a single systems-level account of why comparable exercise stimuli can produce markedly different long-term physiological outcomes. Section 3 examines the empirical and methodological requirements for testing this framework, including candidate biomarkers, appropriate sampling windows, and study designs capable of distinguishing sleep-gated consolidation from the other determinants of Adaptive Consolidation Efficiency identified above.

## 3. Testable Predictions and an Operational Framework for SGA and ACE

### 3.1. Rationale and Scope of Operationalization

The value of a conceptual framework depends on whether it generates predictions that can, in principle, be shown to be wrong. The evidence reviewed in Section 2 was not uniformly positive: sleep restriction reliably altered some consolidation domains (protein-synthetic anabolism, motor-memory consolidation) but only conditionally altered others (the post-exercise immune response, the intermediate phase of autonomic recovery), and several well-designed studies returned clear nulls. This pattern is the central design constraint for this study. A prediction that simply states “sleep restriction impairs exercise adaptation” would be neither novel nor falsifiable because it does not specify the conditions under which the effect should and should not appear. Accordingly, every prediction proposed below specifies not only an expected direction of effect but also the boundary conditions required for that effect to be detectable and an explicit criterion under which the prediction would be considered disconfirmed.

Consistent with Section 2.3 and Section 2.4, this section does not propose a composite SGA score or ACE index. Collapsing multiple physiological domains with different evidence strengths, boundary conditions, and, in several cases, genuinely null intermediate-window findings into a single numeric threshold would misrepresent the state of the evidence reviewed in Section 2 and would imply a level of measurement precision that has not been demonstrated. Instead, the framework was operationalized as a structured set of domain-specific, falsifiable predictions (Section 3.2), a tiered candidate biomarker panel tied to the specific readouts and sampling windows already validated in the literature reviewed in Section 2 (Section 3.3), and minimum study-design requirements for testing the SGA and ACE components of the model separately (Section 3.4 and Section 3.5).

### 3.2. Testable Predictions (P1–P7)

Predictions P1–P5 concern the Sleep-Gated Adaptation component of the framework and are organized by consolidation domain, in decreasing order of the strength of existing mechanistic and causal evidence established in Section 2.3. Predictions P6–P7 concern the Adaptive Consolidation Efficiency component and address whether the multi-determinant structure proposed in Section 2.4 provides explanatory value beyond the sleep state alone.

A qualification applies to all seven predictions that follow. P1–P7 are stated in qualitative terms (for example, ‘adequately powered,’ ‘substantial fraction,’ ‘partially but not fully’) rather than with pre-specified numerical thresholds. As worded, they should be understood as falsifiable in principle rather than as rigorously falsifiable in the strict sense of that term: because the qualitative terms are not numerically anchored, a given result could in principle be argued post hoc to be consistent with a prediction even when it was not clearly anticipated by it. Rigorous falsification of P1–P7 requires that each qualitative term be replaced with a numerical threshold, derived from a formal power analysis grounded in effect sizes from the primary literature cited below, and specified prospectively in a pre-registered protocol before data collection. We present the predictions in this qualitative form because we judge it more transparent to state the intended direction and logic of each prediction than to fabricate numerical thresholds that would not themselves be empirically grounded (Section 3.7); however, we state explicitly that P1–P7 should be read as a template for pre-registration rather than as ready-to-test hypotheses in their current form.

**P1—Protein-synthetic and mitochondrial domain (strongest predicted effect, partially exercise-rescueable).** Under sustained sleep restriction (multiple consecutive nights of markedly reduced sleep opportunity), myofibrillar and sarcoplasmic protein fractional synthesis rates and mitochondrial respiratory function will be reduced relative to a matched normal-sleep condition; concurrent high-intensity exercise during the restriction period will partially but not fully normalize these deficits. Falsification: An adequately powered, stable-isotope-based replication finding no reduction in fractional synthesis rate or respiratory function under sustained restriction, or finding that concurrent exercise fully (rather than partially) normalizes these readouts would disconfirm this prediction. Basis: [6,7,8,9].

**P2—Neural and motor memory domain (uniquely sleep-gated, no demonstrated waking substitute).** Retention and functional cortical reorganization following motor-skill acquisition will be measurably greater after an intervening sleep period than after an equivalent waking interval of the same duration, and no waking intervention (including additional practice) will fully substitute this effect. Falsification: A study demonstrating that an equivalent period of wakefulness (with or without additional practice) produces motor-memory consolidation statistically indistinguishable from sleep would disconfirm the claim that this domain is uniquely sleep-gated. Basis: [10].

**P3—Immune domain (conditional on damage load, sleep loss severity, and readout timing).** Sleep restriction measurably amplifies post-exercise interleukin-6, C-reactive protein, and creatine-kinase responses only when three conditions co-occur: a damaging or prolonged exercise stimulus, a total or multi-night sleep insult, and a delayed (approximately 16–48 h) readout window. The same manipulation under brief sleep insult, non-damaging or short-duration exercise, or an early readout window is not expected to produce a detectable interaction. Falsification: A factorial design that satisfies all three high-boundary-condition criteria simultaneously (severe or sustained sleep loss, damaging or prolonged exercise, delayed readout) and finds no interaction—as opposed to a design satisfying only one or two of these criteria—would disconfirm the domain-conditional immune-gating prediction. Basis: [51,52,53,54]; boundary-defining null: [55].

**P4—Autonomic domain (window-dependent: early reactivation and overnight windows, not the multi-hour recovery curve).** Sleep restriction blunts heart rate recovery in the first 30–60 s after maximal exercise and reduces parasympathetic heart rate variability indices measured during subsequent slow-wave sleep, but does not reliably alter the intermediate, multi-hour (approximately 1–6 h) post-exercise heart rate variability recovery curve. Falsification: A well-controlled study finding that sleep restriction fails to alter early (0–60 s) heart-rate recovery or nocturnal slow-wave sleep variability would disconfirm the early window and overnight components of this prediction; conversely, a well-controlled study consistently finding that sleep restriction does alter the intermediate multi-hour recovery curve across independent replications would indicate that the window restriction itself needs revision. Basis: [56,57,58,59,60,64,65,66]; boundary-defining null for the intermediate window: [67].

**P5—Post-exercise transcriptomic program (qualitative reshaping, not merely attenuation).** Sleep restriction combined with exercise will produce a post-exercise skeletal muscle transcriptomic signature that differs qualitatively—in which specific genes are regulated, not only in the magnitude of a shared response—from the signature produced by the same exercise under normal sleep, with low gene-level overlap between conditions. Falsification: A replication finding that the sleep-restricted and normal-sleep transcriptomic responses are highly overlapping (differing mainly in magnitude rather than gene identity) would disconfirm the qualitative-reshaping claim and would instead support a simpler attenuation model. Basis: [49].

**P6—Multi-determinant structure of ACE.** Genetic variation at exercise-response loci, baseline epigenetic status at exercise-responsive regulatory regions, baseline biological characteristics (fitness, sex hormone milieu, age), environmental and lifestyle exposure (including but not limited to sleep history), and the degree of match between individual biology and training prescription will, in combination, explain a greater share of variance in a defined training-adaptation outcome than any single determinant considered alone—and this combined explanatory contribution will persist after statistically accounting for sleep-restriction exposure during the study window. Falsification: A longitudinal design in which sleep-restriction exposure alone accounts for the substantial majority of outcome variance, with the remaining determinants contributing negligible independent explanatory power once sleep exposure is accounted for, would indicate that the multi-determinant ACE construct adds little beyond the SGA gate itself. Basis: [11,12,13,14,15,16,17,18,69,70,71,72].

**P7—Prescription match as a removable component of an apparent low ACE.** Individuals classified as low responders to one training modality will show a normalized (non-low-responder) profile when reassigned to a different, better-matched training modality within the same individual. Falsification: a crossover design in which a substantial fraction of individuals remain low responders across multiple, meaningfully different training modalities would indicate that intrinsic, modality-independent variance dominates the apparent non-response, rather than prescription mismatch as proposed here. Basis [17,18]. To avoid circularity in the definition of a ‘better-matched’ modality, modality match must be established independently of the outcome under test—for example, via a baseline physiological or genetic marker plausibly linked to modality-specific response (such as the fiber-type or metabolic-phenotype markers discussed in Section 2.4)—rather than being inferred from the fact that a participant subsequently responded to it; a design in which modality match is defined only post hoc by outcome would not constitute an adequate test of this prediction.

Table 2 consolidates predictions P1–P7 for rapid comparison; the full statement, rationale, and falsification logic for each prediction are given in the preceding paragraphs and should be consulted before any prediction is used to design a study.

### 3.3. Candidate Biomarker Panel and Sampling Windows

Table 3 summarizes the candidate readouts for each domain addressed by predictions P1–P5, together with the specimen or method, the sampling window over which an effect is expected to be detectable, and the evidence already establishing that window in Section 2. The panel is deliberately restricted to readouts and windows already demonstrated in the literature synthesized above, rather than proposing untested assay combinations; where a domain’s evidence base derives predominantly from animal or cellular models (as flagged in Section 2.2.1 and Section 2.2.2, and Section 2.4), this is noted so that the panel is not mistaken for a validated human protocol.

### 3.4. Minimum Design Requirements for Testing the SGA Component

The minimum factorial design requirements for testing the SGA component of the framework (predictions P1–P5) are summarized in Box 3.

Box 3 **Factorial Requirements for Testing the SGA Graded-Gate Hypothesis (P1–P5).**

**Rationale**—Section 2.3 established that positive sleep × exercise interactions cluster under specific combinations of sleep loss severity, exercise damage/duration, and readout timing, while null findings cluster under complementary combinations. A design that does not orthogonally vary these three factors cannot distinguish the true absence of sleep dependence from a boundary-condition mismatch.

**Factor 1: Sleep condition**—at minimum, three arms: normal sleep (control), multi-night partial restriction (e.g., ≤5 nights at ≤4 h time-in-bed), and total sleep deprivation (single night), characterized by polysomnography or, where infeasible, validated actigraphy with sleep-stage estimation.

**Factor 2: Exercise stimulus**—at minimum, two arms: a damaging or prolonged stimulus (e.g., standardized eccentric-contraction protocol or exercise duration ≥ 2 h) and a non-damaging, short-duration stimulus (e.g., submaximal steady-state exercise ≤ 45 min), matched where possible for relative intensity.

**Factor 3: Readout timing**—pre-registered sampling at early (0–15 min), intermediate (1–6 h), and delayed (16–48 h) windows, selected before data collection rather than post hoc, given the documented sensitivity of this literature to window selection (Section 2.3).

**Minimum reporting**—objective sleep-architecture data (not only time-in-bed), a standardized and quantified damage/duration metric for the exercise stimulus, and pre-specified rather than exploratory readout windows, so that a null result can be interpreted against the boundary-condition pattern rather than attributed to an unspecified methodological choice.

### 3.5. Minimum Design Requirements for Testing the ACE Component

The minimum longitudinal design requirements for testing the ACE component of the framework (predictions P6–P7) are summarized in Box 4.

Box 4 **Longitudinal Multi-Determinant Design for Testing ACE (P6–P7).**

**Rationale**—Section 2.4 established that no single determinant (genetic, epigenetic, baseline-biological, environmental, or prescription-related) accounts for the majority of interindividual variability in the training response and that a meaningful share of apparent variability reflects measurement noise rather than biology. Therefore, testing ACE requires a design that measures multiple determinants concurrently within the same individuals, rather than studies that examine one determinant in isolation.

**Baseline phenotyping**—targeted genetic panel at established exercise-response loci; baseline DNA methylation at exercise-responsive regulatory regions; baseline fitness, sex hormone profile, and age.

**Concurrent monitoring**—continuous or repeated-interval sleep tracking (actigraphy at minimum), training-load diaries, and dietary logs across the full intervention window–so that environmental determinants are measured rather than assumed constant.

**Prescription-match control**—a within-subject crossover across at least two meaningfully different training modalities (e.g., endurance and resistance) to separate genuine intrinsic non-response from modality mismatch, following the design logic already validated in the twin-crossover literature (Section 2.4).

**Outcome measurement**—multiple, clinically or functionally relevant training-adaptation traits assessed in parallel, rather than a single composite outcome, consistent with the trait-specific rather than global nature of “response” established in Section 2.4; and repeated-measures or replicate testing sufficient to distinguish genuine low response from measurement noise.

### 3.6. Falsification Criteria

Consolidating the falsification criteria specified for each prediction, the SGA–ACE framework as a whole would be substantially weakened by any of the following outcomes: (i) a well-powered factorial design satisfying the high-boundary-condition cell for the immune or autonomic domains (severe/sustained sleep loss, damaging/prolonged exercise, delayed readout) that nonetheless finds no interaction, which would indicate that the domain-conditional gating pattern identified in Section 2 does not hold under its own best-case detection conditions; (ii) a demonstration that waking intervals fully substitute for sleep in motor-memory consolidation, removing the one domain currently considered uniquely sleep-gated; (iii) a longitudinal ACE study in which sleep-restriction exposure alone accounts for the substantial majority of outcome variance, rendering the additional determinants proposed in Section 2.4 explanatorily redundant; and (iv) a multi-modality crossover in which a substantial fraction of individuals remain low responders regardless of training modality, which would shift the balance of evidence away from prescription mismatch and toward a more fixed, intrinsic non-response phenotype than Section 2.4 currently supports. None of these outcomes would necessarily invalidate the broader distinction between transient signaling and durable consolidation established in Section 2.1 and Section 2.2; they would specifically narrow or revise the sleep-gating and multi-determinant components of this model.

### 3.7. Limitations of the Operational Framework

Several limitations constrain how the predictions above should be interpreted and applied. First, and most importantly for translational relevance, the great majority of the positive sleep × exercise interaction evidence underlying P1–P5 derives from acute or severe experimental sleep-loss models—total deprivation or multiple consecutive nights of markedly restricted time-in-bed—rather than from the chronic, moderate sleep debt more typical of real-world training environments. The predictions above should therefore be read as applying most directly to acute or severe sleep-loss scenarios; their extension to cumulative, moderate sleep debt across a training macrocycle is plausible but has not been directly tested by the evidence reviewed in Section 2 and constitutes a distinct, currently untested prediction in its own right.

Second, the causal evidence for a sleep-architecture-to-autonomic-state mechanism (slow-wave-activity manipulation altering heart-rate variability) has been established in a baseline, non-exercise context; its extension to the post-exercise recovery window, proposed in P4, is inferred from convergent but indirect evidence rather than demonstrated by a single study that combines both manipulations. Similarly, the sleep×chromatin-accessibility interaction is untested: Section 2 established that training state reshapes post-exercise chromatin kinetics, but no study reviewed here directly manipulated sleep and measured chromatin accessibility, so this domain is not included among P1–P5 and remains an open question for future operationalization.

Third, several of the mechanistic domains most relevant to ACE—hematopoietic-progenitor epigenetic persistence, central autonomic nuclear neuroplasticity—rest predominantly on rodent or cellular evidence, as already flagged in Section 2.2.1 and Section 2.2.2, and Section 2.4. The corresponding rows in Table 3 should be read as identifying plausible human translational targets rather than validated human assays. As already noted in Section 2.2.1 and Section 2.2.2, these two mechanisms in particular are derived from rodent models; their inclusion in the candidate biomarker panel identifies them as plausible human translational targets, not as findings already demonstrated in humans.

Fourth, consistent with the position taken throughout this review, ACE is not operationalized here as a composite score, and the biomarker panel in Section 3.3 measures its proposed determinants individually rather than producing a unified index. Prematurely aggregating heterogeneous, differently validated readouts into a single number—an approach adopted by some prior frameworks in this literature—would obscure exactly the domain-specific boundary conditions that Section 2 identified as essential to correct interpretation, and is deliberately avoided here.

Fifth, several of the qualitative terms used in Section 3.2 to specify predictions and falsification criteria (for example, ‘adequately powered,’ ‘substantial fraction,’ and ‘most variance’) are not accompanied by pre-specified numerical thresholds. This is a genuine limitation of the current operationalization, not a peripheral caveat: as worded, several of these criteria could in principle be interpreted post hoc in a manner that shifts to accommodate an unexpected result, and P1–P7 should therefore not be described as rigorously falsifiable in their present form (see the qualification introducing Section 3.2). We did not consider it appropriate to invent specific quantitative thresholds without a formal power analysis grounded in effect sizes from the primary studies cited, which lies beyond the scope of a conceptual review; however, translating each prediction in Section 3.2 into a pre-registered protocol with numerically specified thresholds is a necessary step before any of these predictions could be considered to have been rigorously tested, and we state this explicitly rather than leaving it implicit.

### 3.8. Synthesis

This section translated the conceptual SGA–ACE framework developed in Section 2 into seven falsifiable predictions, a biomarker panel restricted to readouts and sampling windows already validated in the literature reviewed, and minimum design requirements capable of distinguishing genuine sleep-gating and multi-determinant efficiency effects from the boundary-condition artifacts that have generated apparently contradictory findings in this field to date. The predictions are deliberately uneven in their expected strength and generality, mirroring the underlying evidence base (Table 1). Whether the framework survives the falsification tests proposed here is an empirical question that lies beyond the scope of the present narrative review; the purpose of this section is to make that question answerable.

## 4. General Discussion

### 4.1. Synthesis: Revisiting the Guiding Research Questions

The preceding sections developed and operationalized a conceptual framework addressing the five guiding questions introduced in Section 1.3. This section synthesizes the evidence bearing on each question in turn and considers what the pattern of evidence—rather than any single finding—indicates about the relationship between exercise-induced signaling, sleep, and interindividual variability.

**Regarding Q1 (the distinction between signaling and adaptation)**, the evidence reviewed in Section 2.1 supports a clear distinction between transient exercise-induced signaling and durable physiological adaptation: molecular signals activated by a single bout resolve within hours to a day, whereas structural and functional adaptations accumulate over weeks [3,4]. Section 2.2 identified the intervening biological processes as a multi-filter transduction cascade—post-translational modification, transcription, protein turnover, coordinated degradation, and chromatin remodeling—operating principally in skeletal muscle [21,22,23,24,26,27], and extended this cascade to two domains not previously integrated into a unified account: immune cell reprogramming, in which a three-wave acute response leaves a durable residue in bone-marrow progenitors, tissue-resident macrophages, and regulatory T cells [32,35,36]; and autonomic remodeling, in which resting heart rate variability and baroreflex sensitivity shift as a trainable, reversible adaptation with an identified neural and cardiac-structural substrate [39,40,41,42,43,44,45]. Adaptive Consolidation is therefore proposed not as a novel mechanism but as the recognition that these domain-specific bodies of literature describe instances of a single underlying biological transition.

**Regarding Q2 (whether sleep is a necessary gate or merely a recovery correlate)**, evidence indicates that sleep functions as a permissive rather than a strictly necessary gate. Several domains, particularly protein-synthetic anabolism and motor-memory consolidation, show clear impairment under sleep restriction that is not fully substitutable by any waking intervention identified in this review [6,7,10]. Simultaneously, concurrent high-intensity exercise partially rescues several of these deficits [7,9], and null findings appear consistently under specific, identifiable conditions rather than unpredictably [55,67]. This pattern is inconsistent with a strict all-or-none switch and is more consistent with a graded, domain-conditional gate, as proposed in the Sleep-Gated Adaptation construct.

**Regarding Q3 (whether all domains show equivalent sleep dependence)**, the answer is unambiguously no. The eight consolidation domains examined in this review do not provide equivalent evidence of sleep dependence. Protein-synthetic and motor-memory domains show the strongest and most direct evidence; mitochondrial, circadian, and transcriptomic program domains show consistent but less mechanistically direct evidence; and the immune and autonomic domains show evidence that is detectable only under specific, identifiable boundary conditions—damage load, sleep-loss severity and duration, and readout timing—with clear, well-designed null findings outside those conditions [51,52,53,54,55,56,57,58,59,60,61,62,63,64,65,66,67] (summarized in Table 1). This domain-specific gradient is, in our assessment, one of the more practically consequential findings of this synthesis because it plausibly explains why individual studies examining a single domain under a single set of conditions have produced an apparently contradictory literature on whether sleep affects exercise adaptation.

**Regarding Q4 (determinants of interindividual variability)**, no single determinant identified in Section 2.4 accounts for the majority of interindividual variability in the training response. Genetic variation explains a meaningful but partial share of trainability [11,12]; response is trait-specific rather than reflecting a global responder phenotype [13,14]; a substantial fraction of apparent non-response resolves with a change in training modality [17]; epigenetic state at exercise-responsive loci differentiates responders from non-responders [15,16]; and a nontrivial portion of apparent variability reflects measurement noise rather than stable biology [18]. Adaptive Consolidation Efficiency is proposed as an organizing label for this multi-determinant structure, explicitly not as a claim that any single determinant is sufficient to predict an individual’s adaptive trajectory.

**Regarding Q5 (whether the framework can be operationalized)**, Section 3 translated the framework into seven predictions (P1–P7), each specifying not only an expected direction of effect but also the boundary conditions required for detection and an explicit falsification criterion, together with a biomarker panel restricted to readouts and sampling windows already validated in the literature reviewed and minimum factorial and longitudinal design requirements for testing the SGA and ACE components separately. Whether the framework survives these tests is, by design, an empirical question that this narrative review does not address.

### 4.2. Implications for Practice and Future Research

Because the great majority of the sleep-restriction evidence underlying Section 2.3 and Section 3.2 derives from acute total deprivation or severe short-term restriction protocols (Section 3.7), the practical implications discussed below should be understood as most directly applicable to comparably acute or severe sleep loss; their extension to the chronic, moderate sleep insufficiency more typical of everyday athletic populations is plausible but has not been directly tested and should not be assumed. Although this review is conceptual rather than interventional, the domain-conditional pattern identified here carries a provisional practical implication: generic guidance to “prioritize sleep for recovery” may be imprecise because the evidence reviewed indicates that sleep loss most reliably compromises protein-synthetic and neural consolidation, while its effects on immune and autonomic consolidation depend on concurrent training load and damage. Until the predictions proposed in Section 3.2 are empirically tested, this implication should be treated as provisional rather than actionable guidance for training or clinical practice.

A further practical implication concerns how the ACE determinants identified in Section 2.4 might eventually inform individualized training decisions. At present, none of these determinants—genetic variants, epigenetic markers, baseline fitness or hormonal profile, or environmental exposures—has been shown to predict an individual’s adaptive trajectory with sufficient quantitative precision to support a clinical or applied decision rule, and we do not present the framework as being at that stage of readiness. What the evidence in Section 2.4 does support is the specific, falsifiable claim (P6) that these determinants, measured jointly and prospectively, will explain more outcome variance than any single determinant considered alone. Demonstrating this would be the necessary first step toward any quantitative, practice-relevant model of ACE; in its absence, any current attempt to assign a numerical weight to genetic, epigenetic, or lifestyle contributions to an individual’s training response would not be supported by the evidence reviewed here. Should the multi-determinant structure of ACE be empirically supported, a natural next stage of this research would be the development of a practitioner-facing assessment tool combining these baseline determinants; we consider this premature at present, given that ACE has not yet been measured independently of training outcome, and note it here as a direction for future work rather than a contribution of the current review.

The falsification criteria proposed in Section 3.6 identify the studies most likely to be informative next: factorial designs that orthogonally vary sleep condition, exercise damage, and readout timing (Box 3), and longitudinal designs that measure multiple ACE determinants concurrently within the same individuals rather than in isolation (Box 4). Two domains identified as evidentiary gaps in Section 3.7—the sleep× chromatin accessibility interaction and the extension of sleep-loss models from acute or severe restriction to the chronic, moderate restriction more typical of real training environments—represent, in our assessment, the most consequential open questions for establishing the translational relevance of this framework.

### 4.3. Limitations

Several limitations, some of which have already been noted in earlier sections, apply to this manuscript as a whole. First, the literature was identified using a targeted, question-driven strategy via Elicit rather than a PRISMA-based systematic review, as detailed in the Literature Review Strategy and Scope sub-section. No formal risk of bias or quality assessment was applied across the included studies, and the evidence base included a small number of preprints and conference abstracts of provisional status.

We emphasize here, in the Discussion, that the evidence-tier hierarchy presented in Table 1 and referenced throughout Section 2 and Section 3 reflects an informal, criterion-based classification (Section 2.3) rather than a formal evidence-grading approach such as GRADE, and should not be interpreted as equivalent in rigor to a hierarchy derived from a systematic review or meta-analysis.

Second, the evidence base is heterogeneous in species, with several domains—hematopoietic-progenitor epigenetic persistence and central autonomic neuroplasticity—resting substantially on rodent or cellular models, whose direct human translational relevance remains to be established.

Third, and most consequential for the translational claims of this framework, the great majority of the sleep × exercise interaction evidence derives from acute or severe experimental sleep-loss paradigms rather than the chronic, moderate sleep debt typical of real training environments; extension of the SGA and ACE constructs to that context is plausible but remains untested.

Fourth, Sleep-Gated Adaptation and Adaptive Consolidation Efficiency remain, at this stage, conceptual organizing constructs rather than validated mechanisms, biomarkers, or indices; the entire framework awaits the empirical testing outlined in Section 3 of this review.

## 5. Conclusions

This review set out to address a specific conceptual gap: three bodies of literature—exercise-induced molecular signaling, sleep-dependent physiological recovery, and interindividual variability in training response—have developed largely independently, despite each describing a stage or determinant of the same underlying process. Three conclusions can be drawn from the synthesis presented here.

First, exercise-induced adaptation is more accurately described as a two-stage process than as a direct consequence of molecular signaling: transient signals generated during and immediately after exercise constitute adaptive information, and a separate, multi-filter biological transition, Adaptive Consolidation, determines whether that information becomes a durable physiological change. This transition spans post-translational, transcriptional, translational, degradative, chromatin-level, immune, and autonomic domains, extending the scope of this concept beyond the muscle-centered signaling cascades that have dominated previous mechanistic accounts.

Second, sleep is best understood as a graded, domain-conditional gate in this consolidation process rather than as either a uniformly necessary requirement or a generic recovery input equivalent to other recovery modalities. The strength of sleep dependence varies systematically across consolidation domains and, for several domains, is detectable only under specific combinations of exercise damage, sleep-loss severity, and measurement timing, a pattern that plausibly explains much of the apparent inconsistency in the existing sleep-and-exercise literature.

Third, the marked interindividual variability long documented in the exercise-training literature is unlikely to be resolved by any single determinant of exercise training adaptation. Genetic, epigenetic, baseline biological, environmental, and training prescription-related factors each contribute measurable but partial explanatory power, motivating Adaptive Consolidation Efficiency as an explicitly multi-determinant, non-quantified organizing construct rather than a composite score.

Taken together, the Sleep-Gated Adaptation and Adaptive Consolidation Efficiency constructs proposed here are offered as testable hypotheses, not as established mechanisms. Their value will be determined by whether the seven falsifiable predictions and associated study designs proposed in Section 3 are ultimately supported or refuted by future empirical studies.

## Figures and Tables

**Table 1:** Evidence gradient for sleep dependence across the eight consolidation domains introduced in Figure 1. GH, growth hormone; IGF-1, insulin-like growth factor 1; HIIE, high-intensity interval exercise; SWS, slow-wave sleep; HRV, heart-rate variability; IL-6, interleukin-6; CRP, C-reactive protein; CK, creatine kinase. Tiers reflect the strength and directness of currently available evidence, not a claim about the biological importance of a domain; a domain in the “conditional” tier may still be substantially affected by sleep loss under the boundary conditions specified.

Domain	Evidence Tier	Detectability/Boundary Conditions	Basis
Protein-synthetic anabolism (GH–testosterone–IGF-1; myofibrillar & sarcoplasmic synthesis)	Strongest	Reliably reduced under sustained restriction; partially rescued by concurrent HIIE	[5,6,7,8]
Neural/motor-memory consolidation	Strongest	No demonstrated waking substitute at any tested duration	[10]
Autonomic—early-window reactivation & nocturnal SWS-phase HRV	Strongest	Causal evidence via slow-wave-activity manipulation; blunted by sleep restriction	[56,57,58,59,60,64,65,66]
Mitochondrial respiratory adaptation	Consistent	Reduced under sustained restriction; partially rescued by concurrent HIIE	[9]
Circadian amplitude/clock alignment	Consistent	Diurnal rhythm amplitude blunted by restriction	[9,47]
Post-exercise transcriptomic program (qualitative shape)	Consistent	Low gene-level overlap between rested and restricted conditions	[49]
Immune response (IL-6, CRP, CK)	Conditional	Detectable only with damaging/prolonged exercise + severe/sustained sleep loss + delayed readout	[51,52,53,54]; null: [55]
Autonomic—intermediate, multi-hour recovery curve	Conditional	No interaction detected in the best-controlled available test	null: [67]

**Table 2:** Summary of falsifiable predictions P1–P7. FSR, fractional synthesis rate; HIIE, high-intensity interval exercise; IL-6, interleukin-6; CRP, C-reactive protein; CK, creatine kinase; SWS, slow-wave sleep; HRV, heart-rate variability; PSG, polysomnography; RNA-seq, RNA sequencing; ACE, Adaptive Consolidation Efficiency; ↑, increase; ↓, decrease; >, greater than; ±, with or without. This table is a compressed reference; falsification of any single prediction narrows or revises the corresponding component of the SGA–ACE framework rather than invalidating the framework as a whole (Section 3.6).

ID	Domain	Expected Pattern	Detection Requires	Falsification Criterion	Basis
P1	Protein-synthetic & mitochondrial	↓ Myofibrillar/sarcoplasmic FSR and mitochondrial respiratory function under sustained restriction; partially rescued by concurrent HIIE	Multiple consecutive nights of restriction; stable-isotope/respirometry measures	No reduction found under restriction; or exercise fully (not partially) normalizes deficits	[6,7,8,9]
P2	Neural/motor memory	Sleep > equivalent wake for retention and cortical reorganization; no waking substitute	12 h interval spanning sleep vs. matched wake	Wake (± extra practice) produces consolidation statistically indistinguishable from sleep	[10]
P3	Immune	↑ IL-6, CRP, CK only when three conditions co-occur	Damaging/prolonged exercise + total/multi-night sleep loss + delayed (16–48 h) readout	High-boundary-condition design (all three present) finds no interaction	[51,52,53,54]; null: [55]
P4	Autonomic	↓ Early heart-rate recovery (30–60 s) and ↓ nocturnal SWS-phase HRV; NOT the 1–6 h recovery curve	Early-window and overnight-window measurement (PSG or validated HRV)	No change in early/nocturnal windows; or a replicated effect in the 1–6 h window	[56,57,58,59,60,64,65,66]; null: [67]
P5	Post-exercise transcriptome	Qualitative reshaping (different genes regulated), not only a magnitude change	48 h post-exercise, rested vs. restricted comparison, RNA-seq	High gene-level overlap between conditions (differing mainly in magnitude)	[49]
P6	ACE—multi-determinant structure	Genetic + epigenetic + baseline + environmental + prescription match jointly explain more variance than any one alone, even after controlling for sleep	Longitudinal design, concurrent multi-determinant measurement	Sleep exposure alone explains most variance; other determinants add negligible power	[11,12,13,14,15,16,17,18,69,70,71,72]
P7	ACE—prescription match	Low responders on one modality normalize on a better-matched modality	Within-subject crossover across ≥2 meaningfully different modalities	Substantial fraction of individuals remain low responders across multiple modalities	[17,18]

**Table 3:** Candidate biomarker panel for predictions P1–P7. HRV, heart-rate variability; HIIE, high-intensity interval exercise; PSG, polysomnography. Sampling windows and readouts are drawn directly from the studies establishing each domain in Section 2.

Domain	Candidate Readout(s)	Specimen/Method	Expected Sensitive Window	Anchor Refs.
Protein-synthetic anabolism (P1)	Myofibrillar & sarcoplasmic fractional synthesis rate; serum GH, cortisol, testosterone	Stable-isotope infusion; serum immunoassay	Acute (single night) and across 5-night restriction protocols	[5,6,7,8]
Mitochondrial/metabolic (P1)	Mitochondrial respiratory function; glucose tolerance	High-resolution respirometry; oral glucose tolerance/continuous glucose monitoring	5-night restriction protocols, with or without concurrent HIIE	[9]
Neural/motor memory (P2)	Motor-sequence retention; task-related cortical/cerebellar activation	Behavioral retention testing; functional imaging	12 h interval spanning sleep vs. equivalent wake	[10]
Immune (P3)	Plasma IL-6, C-reactive protein, creatine kinase	Venous blood, serial sampling	16 h and 48 h post-exercise, only under damaging/prolonged exercise + total or multi-night sleep loss	[51,52,53,54,55]
Autonomic—early window (P4)	Heart-rate recovery at 30–60 s post-maximal exercise; early post-exercise HRV	ECG/validated HRV monitor	0–15 min post-exercise	[64,65,66]
Autonomic—nocturnal window (P4)	Slow-wave-sleep-phase HF-HRV	Polysomnography + concurrent HRV	Night(s) following exercise, scored by sleep stage	[56,57,58,59,60]
Autonomic—intermediate window (P4, expected null)	Multi-hour post-exercise HRV recovery curve	ECG/validated HRV monitor	1–6 h post-exercise	[67]
Post-exercise transcriptome (P5)	Whole-transcriptome skeletal-muscle response	Muscle biopsy, RNA-seq	48 h post-exercise, rested vs. sleep-restricted	[49]
ACE—genetic	Targeted SNP panel at exercise-response loci (e.g., *ACSL1* and related HERITAGE loci)	Genotyping	Baseline (fixed trait)	[11,12]
ACE—epigenetic	DNA methylation at exercise-responsive regulatory regions (e.g., *PGC-1α* promoter)	Targeted bisulfite sequencing	Baseline and post-training	[15,16]
ACE—environmental/lifestyle	Sleep duration and architecture; training load; dietary intake	Actigraphy or PSG; training diary; dietary log	Continuous across the study window	[49,72]
ACE—prescription match	Cross-modality response profile	Crossover training design (e.g., endurance vs. resistance)	Across sequential training blocks	[17]

## Data Availability

No new data were created or analyzed in this study. Data sharing is not applicable to this article.

## Abbreviations

The following abbreviations are used in this manuscript:

ACE	Adaptive Consolidation Efficiency
ACSL1	Acyl-CoA Synthetase Long-Chain Family Member 1
ADP	Adenosine Diphosphate
AMP	Adenosine Monophosphate
AMPK	AMP-Activated Protein Kinase
ATF2	Activating Transcription Factor 2
ATP	Adenosine Triphosphate
BMAL1	Brain and Muscle ARNT-Like 1
CaMKII	Calcium/Calmodulin-Dependent Protein Kinase II
CD14/CD16	Cluster of Differentiation 14/16
COXIV	Cytochrome c Oxidase Subunit IV
CRP	C-Reactive Protein
DNA	Deoxyribonucleic Acid
ECG	Electrocardiogram
FASEB	Federation of American Societies for Experimental Biology
GH	Growth Hormone
GRADE	Grading of Recommendations Assessment, Development and Evaluation
HDL	High-Density Lipoprotein
HERITAGE	HEalth, RIsk factors, exercise Training And GEnetics (Family Study)
HF	High Frequency (heart-rate-variability spectral band)
HIIE	High-Intensity Interval Exercise
HO-1	Heme Oxygenase 1
HRV	Heart-Rate Variability
IGF-1	Insulin-Like Growth Factor 1
IL-6	Interleukin-6
LDL	Low-Density Lipoprotein
MAPK	Mitogen-Activated Protein Kinase
MEF2	Myocyte Enhancer Factor 2
mTOR/mTORC1	Mechanistic Target of Rapamycin/Complex 1
NFAT1	Nuclear Factor of Activated T-Cells 1
NRF-1	Nuclear Respiratory Factor 1
PDK4	Pyruvate Dehydrogenase Kinase 4
PGC-1α	Peroxisome Proliferator-Activated Receptor Gamma Coactivator-1 Alpha
PRISMA	Preferred Reporting Items for Systematic Reviews and Meta-Analyses
PSG	Polysomnography
RMSSD	Root Mean Square of Successive Differences (heart-rate-variability index)
RNA	Ribonucleic Acid
SDNN	Standard Deviation of NN (Normal-to-Normal) Intervals
SGA	Sleep-Gated Adaptation
SNP	Single-Nucleotide Polymorphism
TFAM	Mitochondrial Transcription Factor A
UCP3	Uncoupling Protein 3
Ulk1	Unc-51-Like Autophagy Activating Kinase 1
VO_2_max/VO_2_peak	Maximal/Peak Oxygen Uptake
